# HOF•CH_3_CN—The Most Potent Oxygen Transfer Agent for a Large Variety of Organic Molecules

**DOI:** 10.3390/molecules30061248

**Published:** 2025-03-11

**Authors:** Shlomo Rozen

**Affiliations:** School of Chemistry, Tel Aviv University, Tel Aviv 69978, Israel; rozens@tauex.tau.ac.il

**Keywords:** oxygen transfer, fluorine, HOF•CH3CN, “impossible” oxygenations

## Abstract

The complex of hypofluorous acid with acetonitrile—HOF•CH_3_CN—is the only substance possessing a truly electrophilic oxygen. This fact makes it the only tool suitable for transferring oxygen atoms to sites that are not accessible to this vital element. We will review here most of the known organic reactions with this complex, which is easily made by bubbling dilute fluorine through aqueous acetonitrile. The reactions of HOF•CH_3_CN with double bonds produce epoxides in a matter of minutes at room temperature, even when the olefin is electron-depleted and cannot be epoxidized by any other means. The electrophilic oxygen can also substitute deactivated tertiary C-H bonds via electrophilic substitution, proceeding with full retention of configuration. Using this complex enables transferring oxygen atoms to a carbonyl and oxidizing alcohols and ethers to ketones. The latter could be oxidized to esters via the Baeyer–Villiger reaction, proving once again the validity of the original Baeyer mechanism. Azines are usually avoided as protecting groups for carbonyl since their removal is problematic. HOF•CH_3_CN solves this problem, as it is very effective in recreating carbonyls from the respective azines. A bonus of the last reaction is the ability to replace the common ^16^O isotope of the carbonyl with the heavier ^17^O or ^18^O in the simplest and cheapest possible way. The reagent can transfer oxygen to most nitrogen-containing molecules. Thus, it turns practically any azide or amine into nitro compounds, including amino acids. This helps to produce novel α-alkylamino acids. It also attaches oxygen atoms to most tertiary nitrogen atoms, including certain aromatic ones, which could not be obtained before. HOF•CH_3_CN was also used to make five-member cyclic poly-NO derivatives, many of them intended to be highly energetic materials. The nucleophilic sulfur atom also reacts very smoothly with the reagent in a wide range of compounds to form sulfone derivatives. While common sulfides are easily converted to sulfones by many orthodox reagents, electron-depleted ones, such as R_f_-S-Ar, can be oxidized to R_f_-SO_2_-Ar only with this reagent. The mild reaction conditions also make it possible to synthesize a whole range of novel episulfones and offer, as a bonus, a very easy way to make S^x^O_2_, x being any isotope variation of oxygen. These mild conditions also helped to oxidize thiophene to thiophen-*S*,*S*-dioxide without the Diels–Alder dimerizations, which usually follow such dioxide formation. The latter reaction was a prelude to a series of preparations of [all]-*S*,*S*-dioxo-oligothiophenes, which are important for the efficient preparation of active layers in field-effect transistors (FETs), as such oligomers are considered to be important for organic semiconductors for light-emitting diodes (LEDs). Several types of these oligothiophenes were prepared, including partly or fully oxygenated ones, star-oligothiophenes, and fused ones. Several [all]-*S*,*S*-dioxo-oligo-thienylenevinylenes were also successfully prepared despite the fact that they also possess carbon–carbon p centers in their molecules. All oxygenated derivatives have been prepared for the first time and have lower HOMO-LUMO gaps compared to their parent compounds. HOF•CH_3_CN was also used to oxidize the surface of the nanoparticles of oligothiophenes, leaving the core of the nanoparticle unchanged. Several highly interesting features have been detected, including their ability to photostimulate the retinal neurons, especially the inner retinal ones. HOF•CH_3_CN was also used on elements other than carbon, such as selenium and phosphor. Various selenides were oxidized to the respective selenodioxide derivatives (not a trivial task), while various phosphines were converted efficiently to the corresponding phosphine oxides.

## 1. Discovery and Properties of HOF and HOF•CH_3_CN

We would like to point out that in the past we have reviewed part of the initial results we had obtained using this agent [1]. In this paper we bring a much broader and updated picture covering all known processes involving HOF•CH_3_CN, discovered by the chemical community as a whole. It is our hope that this review will inspire many to try and attach oxygen atoms at sites of numerous molecules which were considered in the past to be impossible to oxygenate.

Elemental fluorine was first isolated by Moissan in 1886, who was awarded the Nobel Prize for this achievement [2]. The first set of experiments with organic substances ended mainly in their destruction, eventually leading to “boycotting” this element from the huge general synthetic efforts carried out by chemists during the last one and a half centuries. Thus, F_2_ has gradually become “taboo” or, as Appelman gently put it, an “autohypnosis” started to evolve, meaning that once chemists accept some “fact”, most do not bother to confirm it anymore [3].

The author of this review completed his post-doctoral training with Professor Sir Derek Barton (Nobel Laureate), who noticed the lack of interest in this element among the chemical community. His encouragement pushed us to start exploring the use of F_2_ in organic chemistry, and from the start, it was clear that to avoid run-away reactions, the elemental fluorine must be diluted up to 5–15% in inert gases such as N_2_. By doing so, we very quickly started to unveil its huge synthetic potential in electrophilic fluorinations [4], in the fluorination of steroidal enones [5], in the electrophilic substitution of tertiary deactivated hydrogens [6], and much more [7]. Almost immediately, it became clear that the potential of fluorine is much higher than plain fluorination reactions, as it could be of help, for example, in the bromination of deactivated aromatic rings, [8] in the iodination of aromatics [9], as well as in the α-halogenation or etherification of practically any pyridine ring [10]. It also could serve as a radical initiator for the polymerization of perfluoro monomers, [11] turning olefins into fluorohydrins, [12] creating methoxilium species (MeO^+^) for electrophilic methoxylation [13,14], and more. We were also most pleased when we found out that it is an essential tool for constructing the best oxygen transfer agent that organic chemistry has to offer—the easily formed complex HOF•CH_3_CN.

The first credible experiment to produce hypofluorous acid–HOF was carried out by the photolysis of F_2_ and H_2_O at low pressure in a solid N_2_ matrix at 14–20 °K. Raising the temperature or pressure resulted in fast decomposition [15]. Appelman took the subject further and was able to isolate a few mg of HOF by reacting 100 tors of F_2_ with water over a Kel-F tube and sampled the product using MS. He spotted a peak at *m*/*z* = 36, indicating the presence of HOF. This, however, was an intermediate that was readily decomposed by water to produce HF and O_2_ (or, in certain conditions, H_2_O_2_) at temperatures higher than −183 °C [16] or by F_2_ itself (producing F_2_O) [17]. Nevertheless, Appelman was able to determine the melting point of the white HOF solid to be −117 °C and the boiling point somewhat below room temperature [3]. Appelman continued his research on the subject and published the results of the solid HOF Raman spectrum interpreted in terms of a hydrogen-bonded structure consisting of planar zigzag chains [18]. The form of these chains was also confirmed by cryogenic single crystal X-ray [19], and the H-O-F angle was calculated by Pross to be (97°) [20]. Appelman also recorded its ^19^F NMR spectrum (+20 ppm) [21]. From all the above, it was clear that as interesting as HOF might be, it cannot serve as a “real” reagent in organic chemistry because of its very limited availability and its extreme stability issues.

**Scheme 1:**
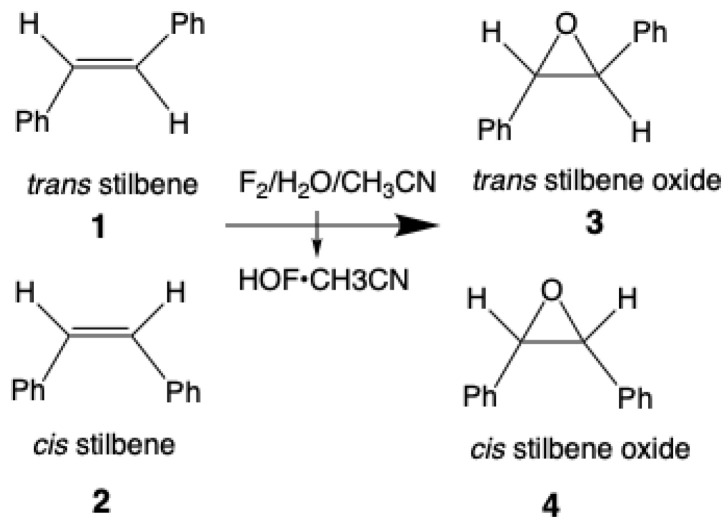
Fast and quantitative epoxidation.

When we worked on fluorination processes with F_2_, we had to rely on reactions between soluble reactants and bubbles of gaseous F_2_/N_2_. We hoped there would be some solvents that could dissolve both the reactants and, to some extent, the fluorine gas, as well. The long list of solvents we tried did not show any oxidative power, meaning no F_2_ was dissolved in them. In one case, however—acetonitrile—a promising lead emerged since a few mmols of oxidative solution were obtained. As we were aware that commercial CH_3_CN is rarely absolutely dry, we took the trouble to remove all traces of water in the hope of increasing fluorine solubility. What happened was just the opposite. No oxidative power was formed. Adding some H_2_O (about 10%) reversed the outcome; the oxidizing power appeared again and was stable at room temperature for a few hours. We reacted this oxidative solution with trans- and cis-stilbenes (**1** and **2**) and noticed that reactions took place practically instantaneously, but the products did not contain any fluorine. This eliminated the possibility that F_2_ was present in the solution. Instead, the corresponding stilbene oxides (**3** and **4**) were formed almost quantitatively [22] (Scheme 1). As we were aware of the Appelman experiments described above, it was clear that fluorine reacted with water, but no free HOF was obtained. It did not take long to realize that the reaction did produce HOF, but this time, it was complexed and stabilized by CH_3_CN to the point that one could comfortably work with its solutions (usually up to 0.6 molar, determined by iodometric titration). These results were highly interesting, as they indicated that this reagent could deliver permanent hydroxylium ion (HO^+^), an almost unknown species in organic chemistry.

**Scheme 2:**
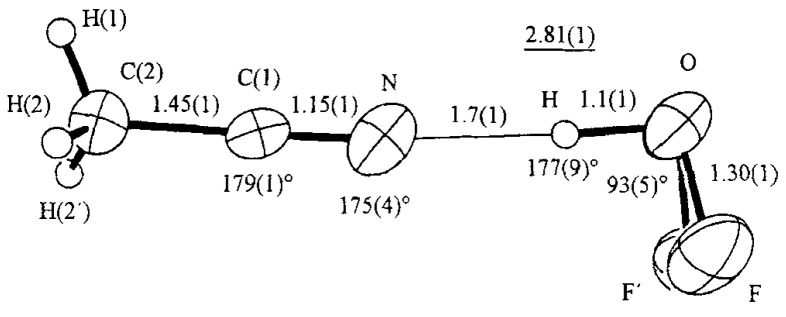
Low-temperature X-ray of the CH_3_CN•HOF complex.

The HOF•CH_3_CN complex soon attracted several physical and theoretical studies. Appelman showed that the complex is a 1:1 hydrogen-bridged entity, in which the HOF proton is most likely bonded to the nitrogen atom of the nitrile. The ^19^F NMR data indicated that the complexation reaction has an equilibrium constant of ca. 3 at room temperature and that the enthalpy of formation of the complex is −14.3 ± 0.5 kJ mol^−1^. The study also showed that strongly decreasing the concentration of the acetonitrile in the CH_3_CN/H_2_O mixture results in a fast decomposition rate of HOF [23]. Soon after, Haas elucidated the structure of the CH_3_CN•HOF complex by low-temperature X-ray, proving the existence of a hydrogen bond between the acetonitrile’s nitrogen atom and the HOF’s hydrogen that is the force for the complexation (Scheme 2) [24].

**Scheme 3:**
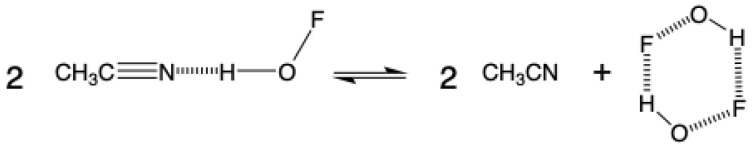
The proposed reactive HOF dimer.

Martin published a theoretical study of the complex structure, vibrational spectroscopy, and the reaction mechanism with olefins. He confirmed our prediction that the charge distribution in the HOF moiety acquires a HO^+^ F^−^ character with partial charges of +0.86 and −0.86 e, respectively, without CH_3_CN and +0.90 and −0.94 e within the complex. Such separation is mainly acquired in the transition state. His calculations also indicated that the hydrogen bond not only stabilizes the reactant but also significantly lowers the epoxidation reaction barrier. Furthermore, the reaction byproduct, HF, can autocatalytically accelerate the reaction [25]. Srnec, who was kind enough to start dubbing the reactions as a “Rozen oxidation”, calculated the stabilization effect of solvents such as water or HF on the HOF (through hydrogen bonding) and found that such a bond with CH_3_CN is, surprisingly, the most stable one. This explains why the role of acetonitrile as an inert solvent is necessary. Srnec also proposed that the reaction mechanism involves a dimer of the reagent (Scheme 3), [26] a proposition that may explain some of the results described later.

## 2. Attaching the Oxygen Atom of HOF•CH_3_CN to Carbon

### 2.1. Constructing Epoxides

**Scheme 4:**
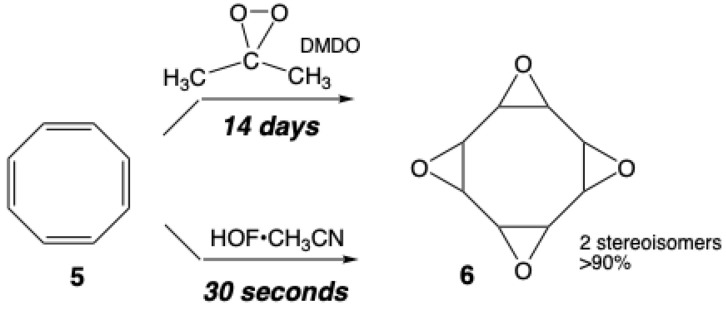
Full epoxidation of cyclooctatetraene.

One of the most popular reactions of olefins is the preparation of epoxides, which for their part, can serve as starting points for many different reactions in pharmacology, material sciences, and many other areas. Usually, these epoxides are made by peroxy reagents such as hydrogen peroxide, m-chloroperbenzoic acid (MCPBA), and, in some more difficult cases, dimethyl dioxirane (Me_2_CO_2_). These reactions usually take hours and high temperatures, but most importantly, they still do not work with many types of double bonds. HOF•CH_3_CN changed the epoxidation field radically. Thus, for example, it was reported that the complete epoxidation of cyclooctatetraene (**5**) with an excess of dimethyldioxirane (DMDO) was achieved only after two weeks (!) [27]. With CH_3_CN•HOF, this long period was reduced to less than 30 s, and the tetra-epoxide (**6**) was obtained in practically quantitative yield (Scheme 4) [28].

**Scheme 5:**
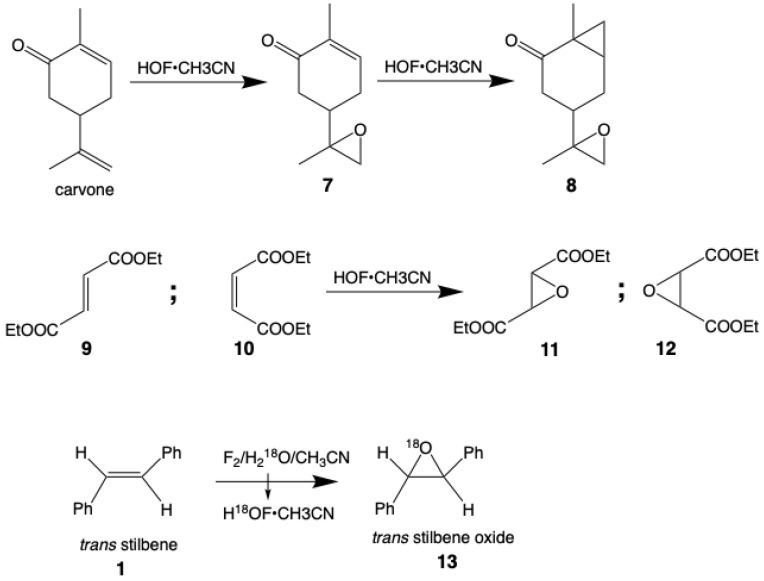
Epoxidation of enones and ^18^O-containing epoxides.

Turning enones to α-keto-epoxides in one step is usually not possible and requires multi-step reactions. There is no such limitation with CH_3_CN•HOF. Thus, the clean mono-epoxidation of carvone (**7**) was achieved after 1 min at 0 °C. When either this monoepoxide or the starting carvone was treated with a 4-fold excess of the reagent for an hour at RT, the diepoxide (**8**) was obtained for the first time at a higher than 50% yield. Even more electron-poor double bonds, such as those in diethyl fumarate (**9**) and maleate (**10**), could be directly epoxidized to **11** and **12,** correspondingly, with a more than 50% yield, although the reaction required harsher conditions than usual (10-fold excess maintained for 12 h. at RT). Maybe this is the place to mention that this method is unique and unprecedentedly easy for constructing epoxides with other oxygen isotopes, such as ^18^O and ^17^O, as demonstrated with **1** producing **13** since the active oxygen originates from the respective water molecule—H_2_^x^O (x = 17, 18, Scheme 5) [29].

**Scheme 6:**
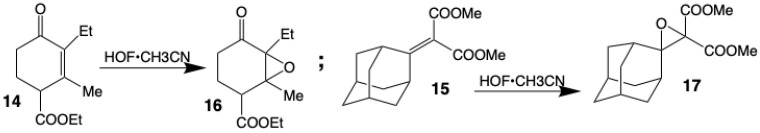
Tetra-substituted epoxides.

Tetra-substituted olefins are particularly difficult to epoxidize. Electron-deficient ones are, in most cases, completely resistant to direct epoxidation. CH_3_CN•HOF has strong electrophilic oxygen and small dimensions, both of which make it most suitable for reacting with such olefins. Thus, 4-(ethoxycarbonyl)-2-ethyl-3-methylcyclohex-2-enone (**14**) and 5-(2-adamantylidene)-2,2-dimethyldicarboxylate (**15**) have never been epoxidized. When treated with CH_3_CN•HOF, both yielded the corresponding epoxide (**16** and **17**) with 85 and 93% yields, respectively (Scheme 6) [30].

**Scheme 7:**
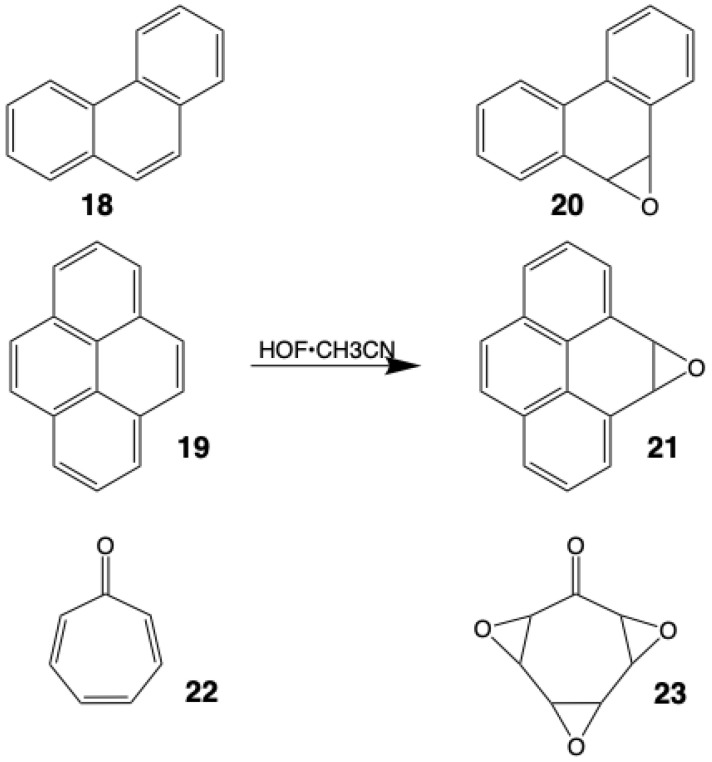
Epoxidation of aromatics.

Arene oxides have been identified as the primary metabolites of polycyclic aromatic compounds, a fact that has stimulated expanding interest in their synthesis and chemistry. Although several preparation routes have been studied, each one has its own limitations. CH_3_CN•HOF seems to be the one that can substitute for all other methods, usually with much better yields and shorter reaction times. Thus, for example, the middle ring of phenanthrene (**18**) or pyrene (**19**) was epoxidized in a matter of seconds to furnish **20** and **21** [31]. Tropone **22**, with its lower density double bonds, has never been fully epoxidized before, but CH_3_CN•HOF required 15 min to epoxidize all three of them (**23**) (Scheme 7) [28].

**Scheme 8:**
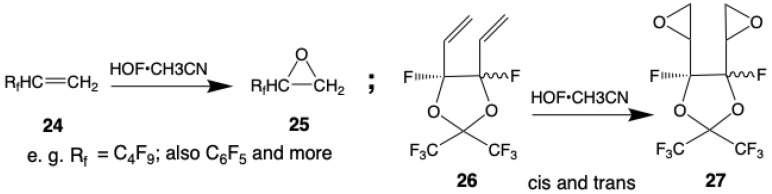
Epoxidation of vinyl-poly-fluoro derivatives.

Fluorine-containing epoxides are important intermediates in the synthesis of surfactants, oligomers, and polymers with special properties. Terminal olefins attached to perfluorinated groups are too deactivated for direct epoxidation, and their epoxides were usually made (if at all) by either using special catalysts or employing indirect methods. For example, epoxidizing the electron-deficient double bond of 3,3,4,4,5,5,6,6,6-nonafluorohexene (**24**) with MCPBA, trifluoroperacetic acid, and by any other orthodox methods was unsuccessful, and only the starting material was recovered. Fortunately, CH_3_CN•HOF was able to provide the respective epoxide (**25**), although high concentrations and long reaction times (of up to 3 h) were needed [32]. This reaction cleared the way for the epoxidation of many other important poly-fluoro olefins, such as fluorinated oxolone (**26**), where the epoxidized (**27**) served as a starting point for the fluoro-polymer Teflon-AF (Scheme 8) [33].

**Scheme 9:**
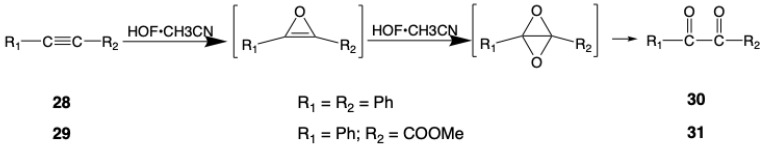
Reactions with acetylenes.

In attempts to transfer electrophilic oxygen to a triple bond, several acetylene derivatives were checked. Unlike olefins, which usually ended with either pure epoxide or a mixture of epoxide and starting material, a mixture of oxygenated materials was formed from the reaction of acetylenes with CH_3_CN•HOF, with the major products being α,β-dione derivatives, apparently obtained via double epoxidation. Among the substrates checked are diphenylacetylene (**28**) and methyl phenyl acetylenic acid (**29**), producing benzil (**30**) and methyl 3-phenyl-2,3-dionepropanoate (**31**) (Scheme 9) [34].

**Scheme 10:**
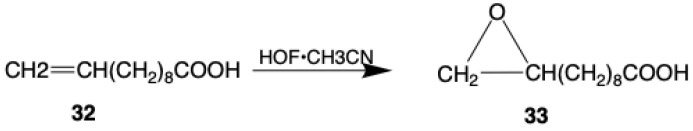
Reactions with free carboxylic acids.

The epoxidation of olefins containing free carboxylic acids is always quite difficult because of solubility issues when reacting with organic epoxidizing reagents. Since the medium of the reactions with CH_3_CN•HOF contains water, it was found that this reagent will epoxidize both olefinic carboxylic acids as well as the respective esters. Thus, for example, Δ^10^-undecanoic acid (**32**) was reacted with two-fold excess CH_3_CN•HOF, resulting in the corresponding epoxide (**33**) (Scheme 10) [35].

This solvent combination encouraged Sandford to develop a continuous flow reaction for creating HOF•MeCN and reacting it fast with substrates, providing an environmentally benign process suitable for large-scale synthesis. It is an atom-efficient system because the only byproduct is aqueous hydrogen fluoride, which could be quenched by any basic substance. Such a process may serve for the large-scale production of many materials relying on olefin epoxidation [36].

### 2.2. Oxidation Reactions

**Scheme 11:**
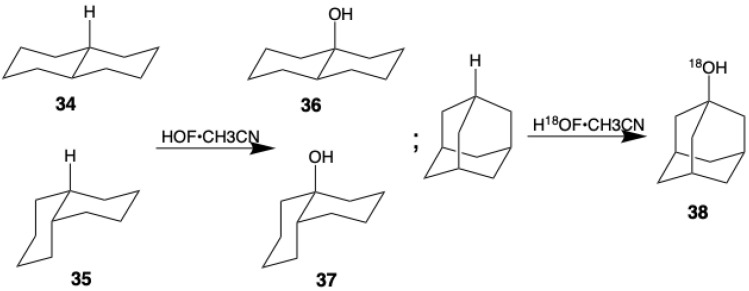
Oxidation of tertiary nonactivated C-H bonds.

Being a strong electrophile, the oxygen atom in the HOF•MeCN can attack the nonactivated, relatively electron-rich, tertiary C-H bonds. Such attacks proceed through non-classical carbonium ions and inevitably result in the full retention of configuration, resembling the reaction of fluorine on similar bonds [6]. Thus, for example, *trans* and *cis*-decalin (**34** and **35**) gave *trans*- and *cis*-9-decalol (**36** and **37**), respectively, with yields higher than 80%. Several additional reactions of this type were reported, including the synthesis of 1-adamantalol with the ^18^O isotope (**38**) with good yield (Scheme 11) [37].

**Scheme 12:**
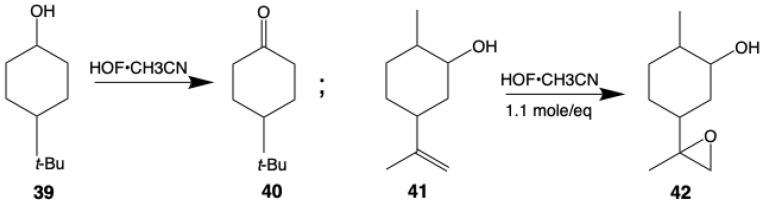
Oxidation of alcohols and competition between alcohols and olefins.

Although HOF•MeCN is very efficient in transferring oxygen atoms to various nucleophilic sites, it is also quite effective in oxidizing reactions, such as turning alcohols to ketones, without any polluting heavy metals or cancerous residues. Thus, for example, 4-*t*-butylcyclohexanol (**39**) was converted to 4-*t*-butylcyclohexanone (**40**) with an 80% yield in less than 10 min. It should be emphasized that if, in addition to the alcohol moiety, some other nucleophilic center is present, such as a double bond, it will take precedence over the hydroxyl oxidation, as evident from the reaction of dihydrocarveol (**41**), which was exclusively epoxidized to (**42**) without any signs of the simultaneous oxidation of the hydroxyl (Scheme 12) [38].

**Scheme 13:**
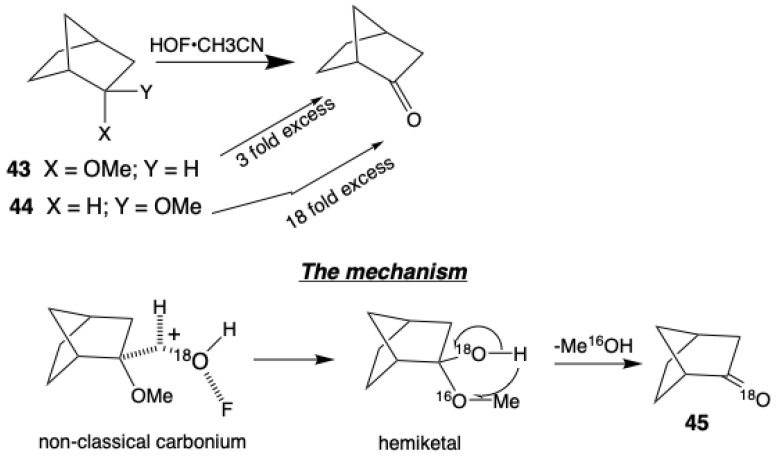
Oxidation of ethers.

As stated above, the mechanism of the oxidation concentrates on attracting the electrophilic oxygen of the reagent to the highest electron density of a C-H bond found in a molecule. In the case of ether, this is the hydrogen α to the oxygenic ether. The reactions of both *endo*- and *exo*-2-norborneol methyl ethers (**43** and **44**) are illuminating. In the case of **43**, the carbon–hydrogen bond α to the ether is relatively easily accessible, and the electrophilic oxygen atom of HOF•CH_3_CN can readily attack it, forming the non-classical carbonium ion. Only a 3-fold excess of reagent was needed to produce the 2-norbornanone (**45**) with an 80% yield. With **44**, the respective C-H bond is in the hindered *endo* position, requiring an 18-fold excess of the reagent to achieve the same results. What is more, when reacting H^18^OF•CH_3_CN with the methyl ether, a carbonyl with ^18^O is exclusively produced. This is explained by producing a hemiketal, which decomposes to the corresponding ketone by the elimination of MeOH [39] (Scheme 13).

**Scheme 14:**
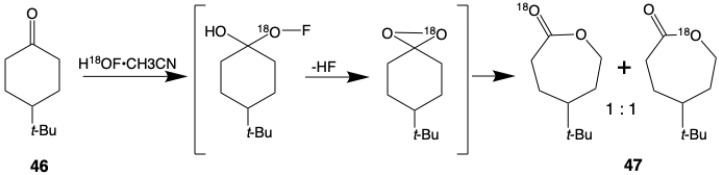
Oxidation of ketones to ester via the dioxirane formation.

Ketones are much less reactive toward HOF•CH_3_CN than alcohols, but this does not mean that they are totally unreactive. When treated with an excess of the reagent for long periods (2–4 h), they are further oxidized to the corresponding esters in a reaction like Baeyer–Villiger oxidation via dioxirane formation. This recalls an interesting “clash” between great chemists. The original mechanism suggested by Baeyer and Villiger themselves for peracids as oxidizers [40] was indeed a dioxirane formation as an intermediate, which inevitably leads to the scrambling of the two oxygen atoms. Doering disputed this mechanism and, in a classical study, proved that the oxygen of the original ketone constitutes the carbonyl in the resulting ester, and only the etheric oxygen atom originates from the peracid [41]. The fact that it is very easy to introduce the ^18^O isotope into the H^18^OF•CH_3_CN reagent was exploited in the reaction with 4-t-butylcyclohexanone (**46**). After 4 h at 0 °C, the ^18^O-containing lactone (**47**) was obtained at a greater than 90% yield. Its Cl mass spectrum showed unequivocally that the ^18^O isotope is equally spread between the carbonyl and the etheric oxygens, bringing back the notion of dioxirane formation [38] (Scheme 14). This is an additional testimony of Baeyer’s great chemical intuition despite his initial wrong mechanism with peracids.

**Scheme 15:**
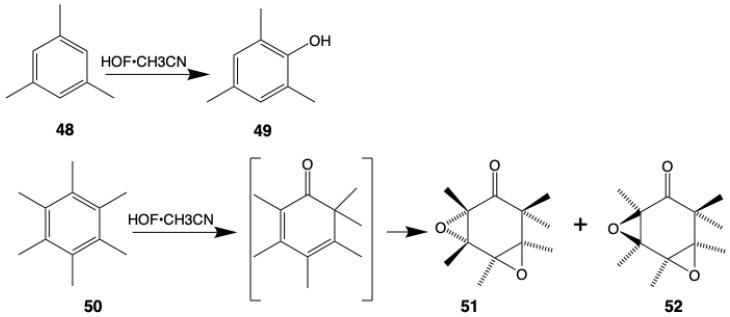
Oxidation of activated aromatics.

HOF•CH_3_CN does not readily react with most aromatic rings, so in general, one should not be much concerned about interfering with a reaction in the presence of such a ring. However, activated aromatics do pose a problem, as they could react if no other more reactive nucleophilic centers are present. Thus, mesitylene (**48**) was oxidized to monohydroxy 2,4,6-trimethylphenol (**49**) with a 45% yield and hexamethylbenzene (**50**) to a 1:1 diastereomeric mixture of the ketodiepoxides (**51**) and (**52**) with a 50% yield. In most such extreme cases, a full conversion should be avoided to minimize tar formation [42] (Scheme 15).

**Scheme 16:**
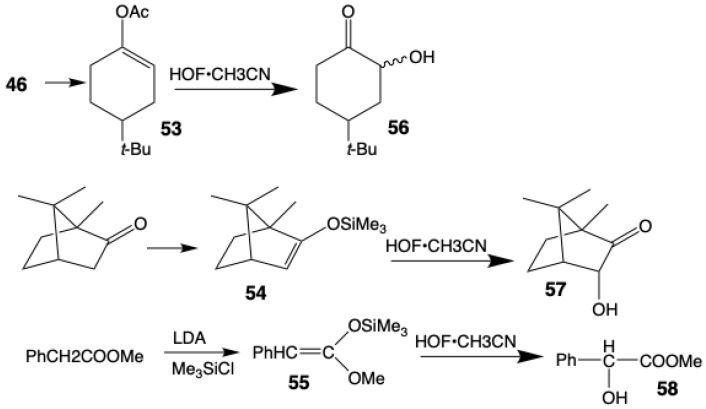
α-Hydroxylations to carbonyls.

The synthesis of α-hydroxy carbonyl derivatives has been of long-standing interest among organic chemists. Several classical methods have appeared in past years employing certain heavy metals, such as Mo and Cr, but naturally, their use has been minimized as much as possible. It is very easy to transform carbonyls to their respective enol derivatives, turning the α-position of the carbonyl into a good nucleophile. Since HOF•CH_3_CN has a strong electrophilic oxygen atom, it can, and does, efficiently react with this electron-rich site to produce the desired α-hydroxy carbonyl derivative in a matter of minutes and usually with higher than 90% yields. Enol acetates, such as the one of 4-*tert*-butylcyclohexanone (**53**) [43], silyl enol ethers (e.g., **54**), or trimethylsilyl ketene acetals such as **55** [44], react equally well to produce the corresponding α-hydroxy derivatives (**56**, **57**, and **58**) (Scheme 16).

**Scheme 17:**
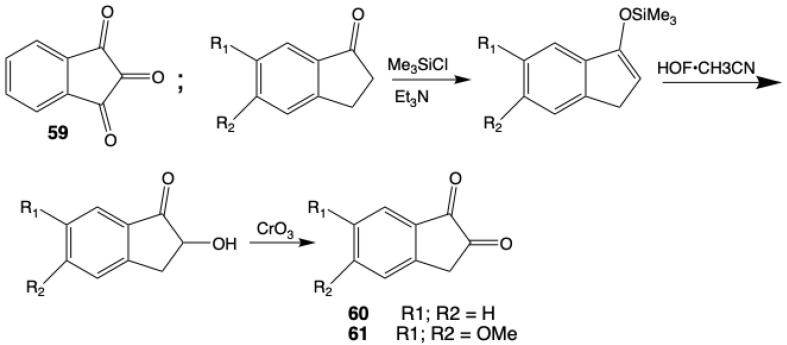
Indanediones for fingerprint visualization.

The above reaction was also found to be useful in forensic science. Ninhydrin (**59**) is an exceptionally useful tool in this branch of science. One of its most notable applications is the visualization of latent fingerprints on paper items. As with many useful procedures, there are always attempts for improvements. It was proposed that 1,2-indandione (**60**) and some of its derivatives, particularly 5,6-dimethoxy-1,2-indandione (**61**), could efficiently visualize latent fingerprints by direct fluorogenic reaction. However, it was challenging to prepare such derivatives, especially **61.** HOF•CH_3_CN solves the problem by reacting with enols of the respective indanones [45] and then oxidizing the α-keto-hydroxyl group (Scheme 17). Indeed, it proved to be an excellent reagent for the visualization of latent fingerprints on paper [46].

**Scheme 18:**
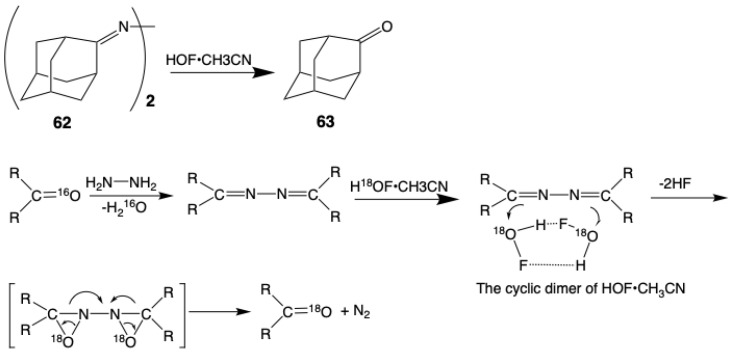
Deprotection of azines by the dimer of HOF•CH_3_CN as suggested in ref. [24] followed by restoration of carbonyls with ^18^O.

There are several ways to protect carbonyls in synthetic chemistry, such as acetals, thioacetals, hydrazones, and the like. Each has a disadvantage, such as sensitivity to acids or bases, difficult removal, or lack of crystallinity, making their purification somewhat tedious. Azines usually form very nice crystals, but since the deprotection step is quite problematic, they are considered to be a dead end. This changes drastically with HOF•CH_3_CN. Reacting many azines obtained from aromatic or aliphatic carbonyls, including adamantylazine (**62**), with a slight excess of HOF•CH_3_CN at 0 °C for a few seconds, offered an excellent way back to the original carbonyl compounds, as demonstrated by the azine **62**, converted to adamantanone (**63**) with a higher than 90% yield. It should be noted that since the original ^16^O of the carbonyl is removed during the azine formation, the latter could be treated with H^18^OF•CH_3_CN, thus opening an easy route to efficiently obtaining carbonyls with the ^18^O isotope [47] (Scheme 18).

**Scheme 19:**
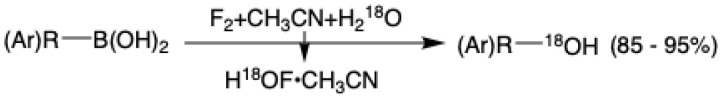
Alcohols with the ^18^O isotope.

As in the case of carbonyls, it is also important to label alcohols with the heavy ^18^O isotope, as they can serve as valuable biological probes. Such studies, however, are often limited by the availability of such labeled alcohols. Here again, H^18^OF•CH_3_CN is the best tool to fulfill this purpose. Alkyl boronic acids are readily available, either commercially or through the corresponding halides. When such acids are treated with the labeled complex made from F_2_, CH_3_CN, and H_2_^18^O, the best and cheapest source of ^18^O atom, the desired ^18^O alcohols were obtained with very good yields based on the H_2_^18^O used [48] (Scheme 19).

## 3. Attaching the Oxygen Atom of HOF•CH_3_CN to Nitrogen

### 3.1. Forming Nitro Groups

**Scheme 20:**
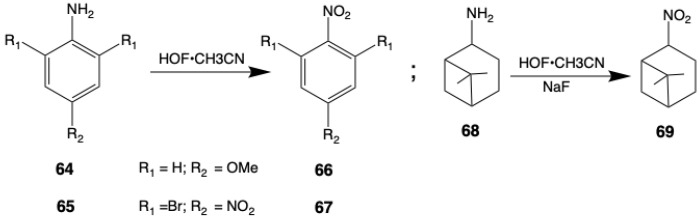
Conversion of amines to nitro derivatives.

The oxidation of various anilines to the corresponding nitro derivatives has been long described. The oxidants were usually peracetic acid, sodium perborate, and dimethyl dioxirane for rings with electron-donating substituents and peroxytrifluoroacetic acid or 98%(!) H_2_O_2_ for the electron-deficient anilines. In general, the yields ranged from 0 to 90%. Each aniline presented its own limitations regarding the pH of the reaction, times, and temperatures. HOF•CH_3_CN oxidizes equally well with various anilines possessing either electron-donating (e.g., 4-methoxy aniline **64**) or electron-deficient groups, such as 2,5-dibromo-4-nitro aniline (**65**), to form the corresponding nitro derivatives **66** [49] and **67** [50] in a few minutes with a higher than 80% yield (Scheme 20).

Aliphatic amines are usually more difficult to oxidize than the aromatic ones. There are a few specific methods for this purpose based on various oxidants, including dimethyldioxirane, which was used by Murray [51]. Another approach consists of condensing at least two smaller molecules, one of which already contains the nitro group. However, all these methods require either lengthy multi-step reactions or metal-containing oxidants, which can create disposal problems. Again, as with various anilines, the HOF•CH_3_CN complex meets no resistance in turning the aliphatic amines, such as the bicyclo mirtanylamine (**68**), to the corresponding nitro derivative (**69**) without any undesired rearrangements. It should be noted, however, that during the synthesis of the HOF•CH_3_CN, at least one molecule of HF is also formed, which can react with the free amine (6 to 7 pKa units more basic than the aromatic ones) and produce non-soluble salt, suppressing the reaction. To minimize the effect of the hydrofluoric acid, NaF was added to the HOF•CH_3_CN, which bound most of the HF in the form of the sparingly soluble NaHF_2_ salt, letting the HOF•CH_3_CN react cleanly and efficiently with the free amine [52] (Scheme 20).

**Scheme 21:**
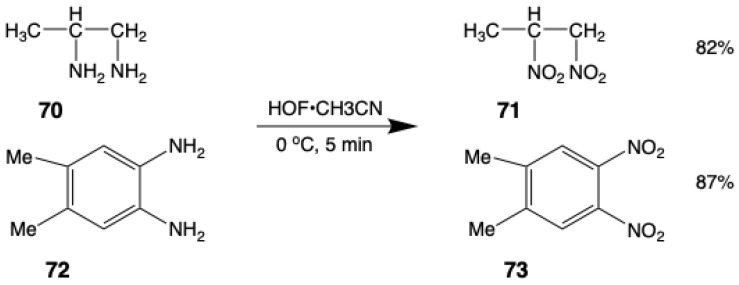
Conversion of 1,2-diamines to the respective 1,2-dinitro derivatives.

While mono-nitrated molecules abound in the chemical literature, this is not the case with vicinal dinitro derivatives. In aliphatic chemistry, two main methods were usually employed: (a) fusing two molecules, each containing a nitrogen residue of a kind, and (b) reacting olefins with the highly toxic dinitrogen tetroxide–N_2_O_4_, resulting, in most cases, in low yields of the 1,2-dinitro products. The conventional oxidation of vicinal diamino groups could not furnish the 1,2-dinitro derivative due to the “push–pull” effect generated during the oxidation process, which tends to break the central C-C bond. The synthesis of 1,2-dinitro aromatic compounds is even more challenging since the nitration of nitroaromatics tends to give mainly m-dinitro derivatives. Since, however, HOF•CH_3_CN is a very strong electrophile, and its reactions are usually conducted at low temperature for a short time, it can react with a second vicinal amino group without damaging the central C-C bond. Thus, it took 3 min to convert 1,2-diaminopropane (**70**) to 1,2-dinitropropane (**71**) with an 82% yield. Quite a few other aliphatic 1,2-diamino compounds were also converted to the respective dinitro derivatives. It should be emphasized that the stereochemistry around the nitrogen-bonded carbons is fully retained. This oxidation also proceeds well with aromatic diamines. 4,5-Dimethyl-1,2-phenylenediamine (**72**) can serve as an example. It reacts with HOF•CH_3_CN for 4 min at −15 °C to produce 4,5-dimethyl-1,2-dinitrobenzene (**73**) with an 87% yield [53] (Scheme 21).

**Scheme 22:**
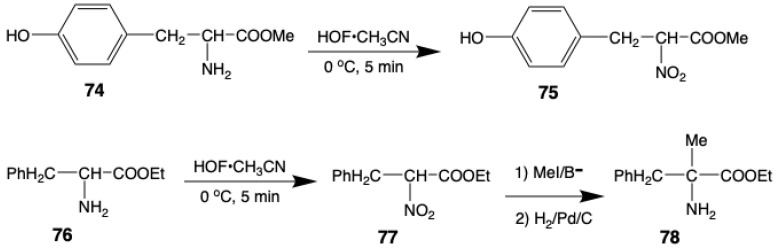
Oxidation of amino acid and the construction of α-alkyl amino acids.

α-Nitro acids are quite useful as a starting point for various syntheses. The problem is that they are not easy to make, and usually, a combination of two fragments, such as nitroacetate and an alkyl group, is needed. The direct oxidation of amino acids, under the relatively harsh conditions generally used, usually leads to ammonia derivatives and nitrogen-free molecules. As we have seen, reactions with HOF•CH_3_CN proceed under mild conditions (room temperature or below and short reaction times), so its reactions with amino acids should not result in the destruction of the whole molecule. Indeed, the results have been the desired α-nitro acids with very good yields. Thus, for example, methyl tyrosine (**74**) was converted at room temperature to methyl 2-nitro-3-(4-phenol)-propionate (**75**) in 5 min and with a 95% yield [54]. This reaction offers a unique way for the synthesis of α-substituted amino acids, which are of great interest (see, for example, [55] and references therein). Phenylalanine ethyl ester (**76**) can serve as an example. Its oxidation leads to ethyl 3-phenyl-2-nitropropionate (**77**), which, in turn, could be easily methylated with MeI (α to both carboxylate and nitro groups). Treating the nitro group with H_2_/Pd/C resulted in the target α-methyl-phenylalanine ethyl ester (**78**) [56] (Scheme 22).

### 3.2. Forming the N-Oxide Moiety—Aliphatics and Six-Membered Rings

**Scheme 23:**
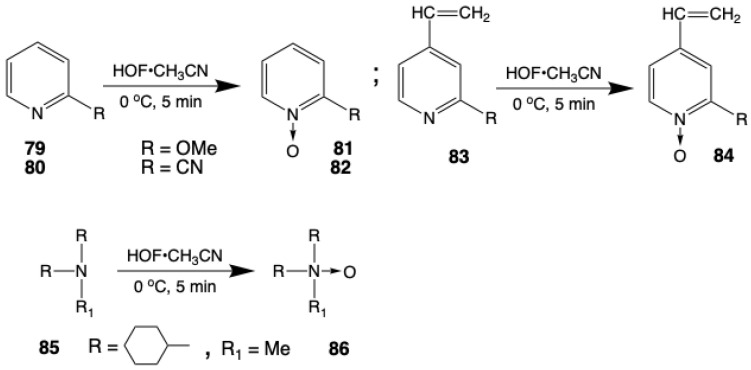
Oxidizing tertiary amines to the corresponding *N*-oxides.

While primary amines were converted to nitro derivatives (see above), tertiary amines of any kind produced the corresponding *N*-oxides with very good yields. In the aromatic field, compounds such as 2-methoxypyridine (**79**) or less basic ones such as 2-cyanopyridine (**80**) produced the corresponding *N*-oxides **81** and **82**. It is of interest to note that if double bonds are present, as in 4-vinylpyridine (**83**), the electrophilic oxygen in HOF•CH_3_CN will attach itself preferably to the tertiary nitrogen, producing 4-vinylpyridineoxide (**84**) with a 70% yield. All aliphatic tertiary amines tested also reacted well, including *N*,*N*-dicyclohexyl-*N*-methylamine (**85**), whose *N*-oxide **86** is used to dissolve cellulose [57] (Scheme 23).

**Scheme 24:**
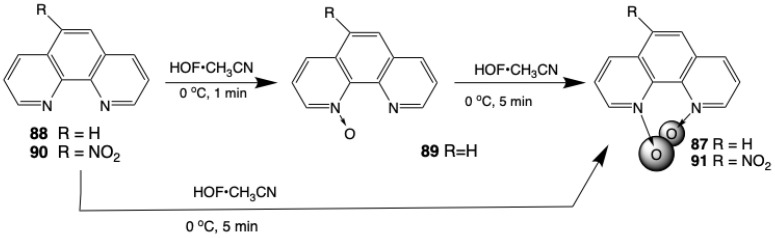
The synthesis of 1,10-phenanthroline-*N*,*N*′-dioxides.

One specific *N*-oxide has an interesting history. During most of the 20th century, people, including Woodward and Corey, tried hard to prepare 1,10-phenanthroline-*N*,*N*′-dioxide (**87**) from the parent phenanthroline (**88**) with little success. Using a variety of oxidants only resulted in the mono *N*-oxide (**89**). A careful examination of the virtual target showed that there is not enough space for two oxygen atoms in the bay area between positions 1 and 10. When, however, the most powerful oxygen transfer agent, HOF•CH_3_CN, was used, **89** was obtained in a minute or two, but using 2.2 equivalents of this reagent for 5 min produced the desired dioxide **87** by taking the aromatic phenanthroline out of planarity, something no other reagent was able to do [58]. The reaction is not limited to **88**, but several additional analogs were also reacted to furnish the respective *N*,*N*′-dioxides, including the electron-deficient 4-nitro-1,10-phenanthroline (**90**), which produced 4-nitro-1,10-phenanthroline-*N*,*N*′-dioxide (**91**), although with a somewhat lower yield of 47% [59] (Scheme 24). Katz recognized the dioxides of type **87** as some of the smallest helicenes that “have within their helical structures possibly useful functional groups that are polar and should ligate to metals” [60].

**Scheme 25:**
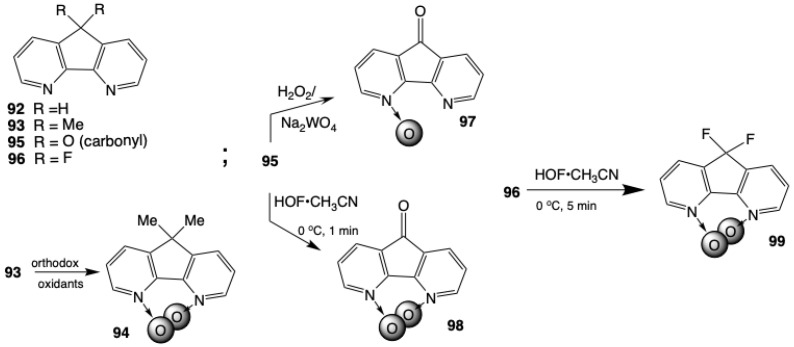
Oxidation of 4,5-diazafluorene derivatives.

Like the 1,10-phenanthroline above, many members of the 4,5-diazafluorene (**92**) family could not be oxidized. There is a patent stating that with two electron-donating groups at the 9 position, as in 9,9-dimethyl-4,5-diazafluorene (**93**), it is possible to make the bis-4,5-*N*,*N*-dioxide **94**. However, no such dioxide could be made when electron-withdrawing groups, such as carbonyl (**95**) or difluoro (**96**), are located at that position, decreasing the electron density on the nitrogen atoms. By treating **95** with a concentrated H_2_O_2_/Na_2_WO_4_ system, only partial oxidation was achieved, forming 4,5-diazafluoren-9-on-4-*N*-oxide (**97**) with only a 7% yield. A large excess of both dimethyl dioxirane (DMDO) and MCPBA did not change the outcome, even after prolonged reaction times. The electrophilic oxygen in HOF•CH_3_CN was able to attack the nitrogen atoms, overcoming their reduced electron density and limited space in the central bay. Reacting a small excess of this powerful oxygen transfer agent with **95** for about a minute resulted in the respective *N*,*N*-dioxide (**98**) with a 95% yield. Applying a 5 mol equivalent of the reagent at room temperature on **96** gave, for the first time, 9,9-difluoro-4,5-diazaflouren-4,5-dioxide (**99**) with a 90% yield [61] (Scheme 25).

**Scheme 26:**
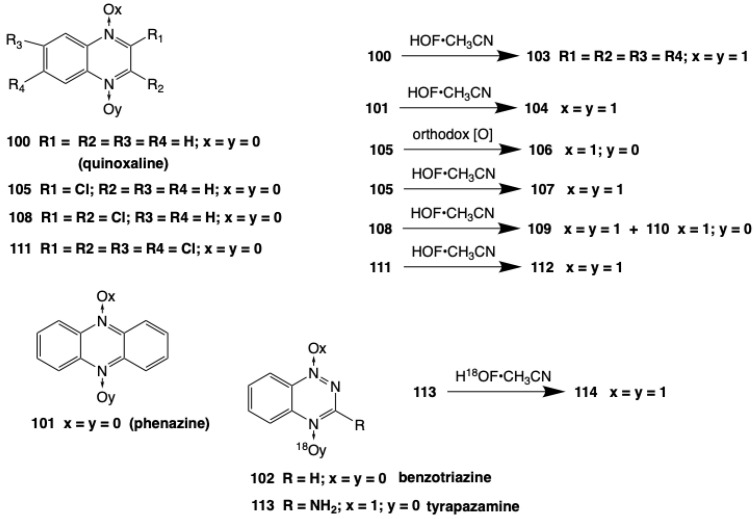
Oxidation of quinoxalines, phenazine, and benzotriazines.

Additional biologically important families of six-membered-diaza aromatics, the quinoxalines (**100**), phenazines (**101**), and benzotriazines (**102**), were also subjected to oxidation. The direct oxidation of quinoxaline itself has been achieved with orthodox oxidants but with low yields and long reaction times. It was not surprising that it took less than 2 min for the HOF•CH_3_CN complex to convert it to quinoxaline *N*,*N*′-dioxide (**103**) with a 95% yield, and similar results were observed for **101** converted to phenazine 5,10-*N*,*N*′-dioxide (**104**). When, however, an electron-withdrawing group such as a single chlorine was present, as in 2-chloroquinoxaline (**105**), reactions with all known oxidizing agents were stopped at the mono-oxide **106** and were not able to continue to the dioxide (**107**). A few seconds of reaction with HOF•CH_3_CN resulted in **106**, and it required a few more minutes to produce the desired *N*,*N*′-dioxide **107** with a higher than 80% yield. It should be mentioned that this dioxide was prepared only once before via total synthesis with a 25% yield [62]. Even adding an additional chlorine atom, as in 2,3-dichloroquinoxaline (**108**), was not a prohibitive factor for the HOF•CH_3_CN, and the dioxide **109** was formed with a 45% yield, along with the corresponding mono-oxide (**110**). In the extreme case of 2,3,6,7-tetrachloroquinoxaline (**111**), the reaction required a 14 mole equivalent excess of the hypofluoro oxidant and 40 min reaction time to make the dioxide (**112**) with a 40% yield [63] (Scheme 26).

Benzotriazine *N*-oxides are an interesting class of experimental anticancer and antibacterial agents. One biological question about the oxygen at *N*-4 was how it would behave in the low-oxygen environment (hypoxic) found in many solid tumors. Would it be eliminated as water or as a hydroxyl radical, which may trigger radical destruction of cancerous DNA? This posed a challenge of introducing ^18^O at this site, in addition to the more common ^16^O found in tirapazamine 1-*N*-oxide (**113**). Obviously, H^18^OF•CH_3_CN was the right tool for that purpose, and indeed, when **113** was reacted with it, the heavy ^18^O atom was attached to the 4-position, forming tirapazamine 1-*N*^16^O,4-*N*^18^O dioxide with a higher than 70% yield (**114**) [64] (Scheme 26).

**Scheme 27:**
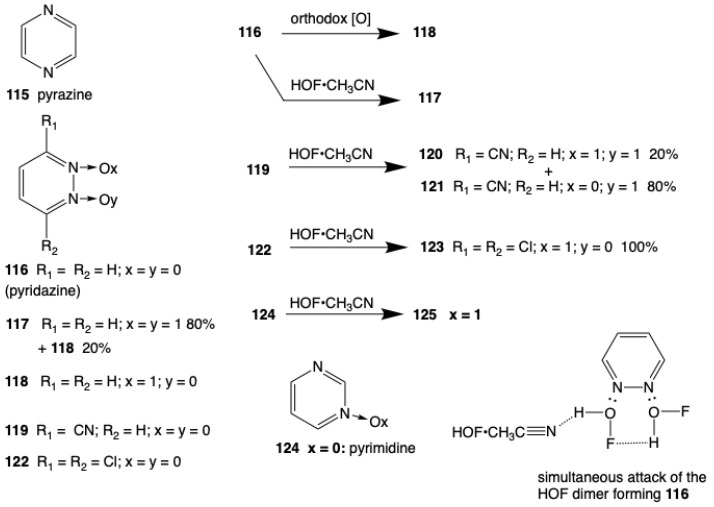
Pyridazine oxidation.

Pyrazine is the basic unit of all the above cases. By itself, (**115**-Scheme 27) and its derivatives (**100**, **101**, **102**) could be oxidized, albeit with difficulty, to form the respective *N*,*N*-dioxides, as we have seen in Scheme 26. The related pyridazine (**116**), however, proved impossible to oxidize with orthodox oxygen transfer agents to form pyridazine *N*,*N*-dioxide (**117**). There were several attempts described in the literature to do that, including treating **116** with boiling 90%(!) H_2_O_2_ for a prolonged reaction time, only resulting in pyridazine mono-oxide (**118**). When, however, HOF•CH_3_CN was reacted with (**116**), the elusive *N*,*N*-dioxopyridazine (**117**) was obtained with an 80% yield almost instantaneously (structure verified by X-ray). The balance of the reaction was the mono-oxide (**118**). Placing electron-withdrawing groups (EWGs) on the ring, such as in 3-cyanopyridazine (**119**), reduced the nitrogen’s basicity, and the yield of the respective dioxide (**120**) dropped to 20%, with the balance being the mono-oxide **121**. More EWGs on the pyridazine skeleton proved to be too much, even for the HOF·CH_3_CN. Thus, the 3,6-dichloropyridazine (**122**) and other similar derivatives only yielded the corresponding mono-oxide (**123**) regardless of how much excess HOF·CH_3_CN was used. Unlike many other cases where mono-oxides of any type could be further oxidized to the dioxides, this was impossible in the case of pyridazines. The reason seems to be the low basicity of the second vicinal nitrogen once an oxygen atom is attached to the first nitrogen. This behavior supports the Srnec hypothesis (see ref. [26]) that the architecture of the oxidizer is a dimer through hydrogen bonding, so it can simultaneously attack the two vicinal nitrogen atoms. Please note that in such a format, these oxygens cannot simultaneously approach the two nitrogen atoms of the pyrimidine (**124**), and indeed, only the mono pyrimidine oxide (**125**) was formed, resisting a second oxidation [65] (Scheme 27).

### 3.3. Forming the N-Oxide Moiety—Five-Membered Rings

**Scheme 28:**
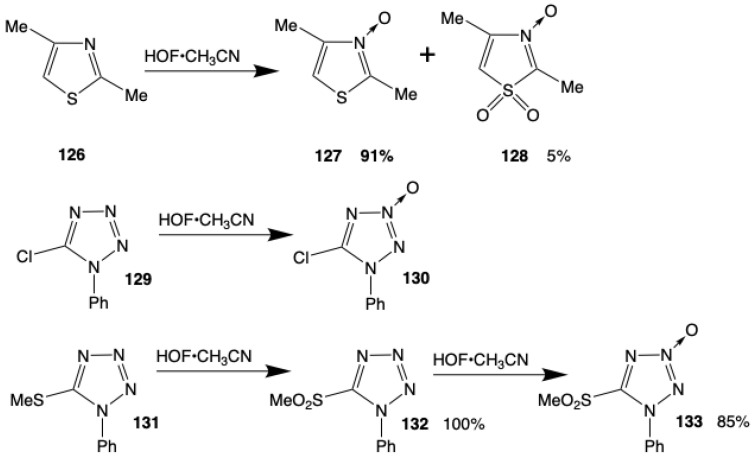
Poly-hetero five-member aromatics.

It was of interest to examine the case where nitrogen and sulfur are found in the same molecule. The immediate question arose: with which nucleophile does the electrophilic HOF•CH_3_CN prefer to react faster? Many times, mixtures resulted. The sulfur in the thiazole ring, however, has two electrons, which are part of the aromaticity. This offered an advantage for the nitrogen atom in competing for the electrophilic oxygen, and indeed, it reacted much faster to form the *N*-oxide. Still, in most cases, the sulfur atom also succeeded in attracting a small amount of the reagent, resulting in the respective *N*,*S*,*S*-trioxide. Thus, the reaction of 2,5-dimethylthiazole (**126**) provided the respective *N*-oxide **127** with a 91% yield (structure also proven by X-ray), along with 5% of the trioxide **128**. Quite a few thiazole derivatives were reported, and all showed similar results [66] (Scheme 28).

A different five-membered heteroaromatic ring is the tetrazole. Although not found in nature, the tetrazole ring appears in a wide range of important products, such as explosives and many drugs. It was agreed by most that the tetrazole ring resists oxidation (see also ref. [2] on “Autohypnosis!”), even when very strong oxidizing agents were employed. As seen by now, HOF•CH_3_CN is in a category by itself when the oxygen transfer process is in question. Thus, for example, while 5-chloro-1-phenyltetrazole (**129**) did not react with 1 mole/eq HOF•CH_3_CN, a 10-fold excess of it completed the reaction, forming the new 5-chloro-1-phenyltetrazole-3*N*-oxide (**130**) with a 95% yield (but with 50% conversion). Several other tetrazole derivatives were prepared using the same procedure. It should be mentioned that unlike the thiazole case, where the nitrogen was more active than the sulfur atom in attracting the electrophilic oxygen (**127** > **128**), the situation was reversed in the tetrazole series, as demonstrated by the oxidation of 5-methylsulfide-1-phenyltetrazole (**131**). When using 5 mole/equiv of the reagent, 5-methylsulfone-1-phenyltetrazole (**132**) was obtained with quantitative yield, emphasizing the resistance of the tetrazole ring toward oxidation. Only by adding an additional 5 mole/equiv of HOF•CH_3_CN to **132** was the desired 5-methylsulfonyl-1-phenyltetrazole-3*N*-oxide (**133**) obtained with an 85% yield [67] (Scheme 28).

### 3.4. Preparation of Energetic Materials

**Scheme 29:**
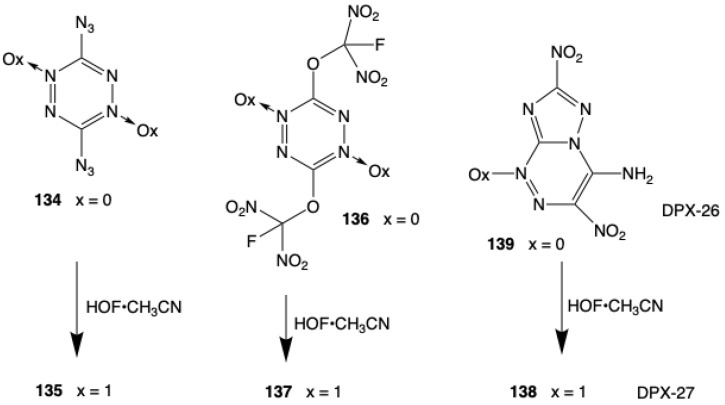
Synthesis of some explosives with HOF•CH_3_CN.

There are several families of highly energetic materials (usually explosives). Some of them are characterized by having the NO moiety, resulting from reactions between heteroaromatic nitrogen and strong oxidants [68]. Since very few, if any, oxygen transfer agents are as efficient as HOF•CH_3_CN, a group in Los Alamos National Labs used it for this very same purpose. They made 3,6-diazido-1,2,4,5-tetrazine (**134**) and tried to react it using peroxytrifluoroacetic acid (PTFA). The reaction was started at 0 °C and warmed to room temperature, but it was found that the oxidation was quite sluggish. The reaction was then performed using 2 equivalents of hypofluorous acid complexed with acetonitrile, and that led to the exclusive production of 3,6-diazido-1,2,4,5-tetrazine 2,5-*N*,*N*-dioxide (**135**) with no evidence of any other oxidation products being formed. It should be mentioned that the detonation velocity of **135** is one of the fastest detonation velocities ever reported [69]. Two additional explosives were also prepared with the aid of HOF•CH_3_CN. The first started with bis-3,6-(2-fluoro-2,2-dinitroethoxy)-1,2,4,5-tetrazine (**136**) and ended up with the 2,5-*N*,*N*-dioxo derivative **37**, with the heat of formation calculated to be −322 kJ/mol, detonation pressure 33.2 GPa, detonation velocity 8.4 kms^−1^, and oxygen balance (OB%) −3.6, very promising data when compared to a conventional explosive such as RDX [70]. The second example was 4-amino-3,7-dinitrotriazolo-[5,1-c][1,2,4]-triazine 4-oxide (DPX-27) (**138**), which was prepared again by oxidation of the parent 4-amino-3,7-dinitrotriazolo-[5,1-c][1,2,4] triazine (DPX-26) (**139**) at −10 °C for several hours and with an 80% yield. Its physical data as explosive, and its performance as such is similar to or better than RDX. It also has the advantage of possessing excellent insensitivity toward destructive mechanical stimuli [71] (Scheme 29).

### 3.5. Miscellaneous Novel Syntheses Involving the Nitrogen Atom

**Scheme 30:**
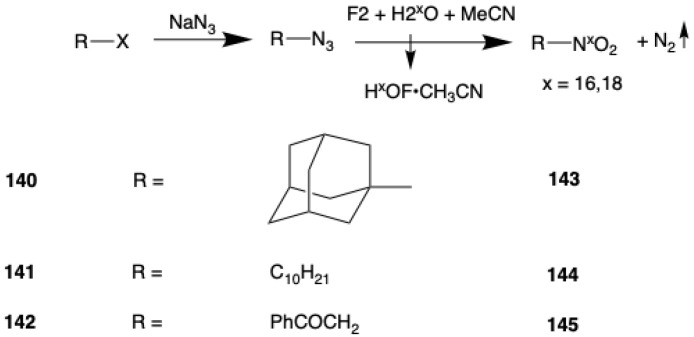
Oxidation of azides to nitro derivatives.

Like Appelman’s “autohypnosis” concept aimed at chemists reluctant to work with elemental fluorine [3], there was also Sharpless’ statement about the “irrational fear” of azides, which he termed “azidophobia” [72]. What these two great scientists meant and complained is that once chemists accept some “fact”, most others do not bother to confirm it…. Still, one of the few ways to introduce nitrogen to many organic molecules via nucleophilic reactions is by using NaN_3_ and the like, followed by reduction to amines. No work has treated azides as candidates for oxidation. As it happens, its reaction with HOF•CH_3_CN proved to be probably the best way to introduce a nitro group wherever the azide group could be introduced. The reaction did not require more than a few seconds at 0 °C to produce the corresponding NO_2_ derivative and, if so desired, to also end up with N^18^O_2_-containing nitro molecules [73]. Quite a few alkyl halides were thus converted to azides, e.g., 1-azidoadamantane (**140**), 1-azidodecane (**141**), and α-azido acetophenone (**142**), to mention just a few. These azides were then treated with HOF•CH_3_CN to produce the corresponding nitro derivatives (**143**–**145**), usually with yields of around 90% [74] (Scheme 30).

**Scheme 31:**
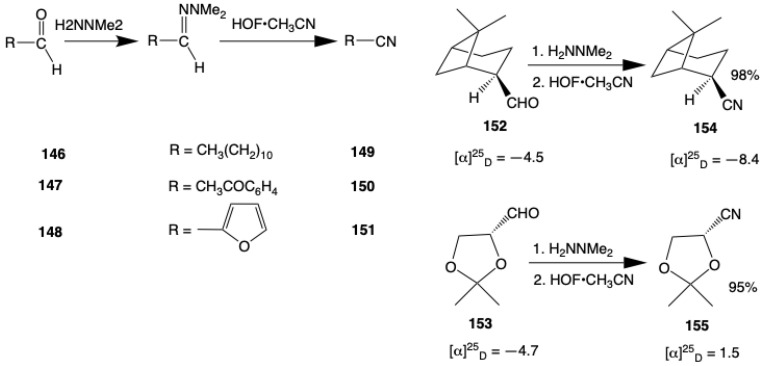
The fast lane from aldehydes to nitriles.

There are traditional ways to convert aldehydes to nitriles. Most start by preparing imines and oxidizing them to create nitriles. Most of these reactions are characterized by using health-hazardous oxidants such as MCPBA or SeO_2_, heavy polluting metals such as molybdenum and tungsten, or expensive metals like rhenium. DMDO or oxone on alumina requires large amounts of organic solvents and lengthy reaction times. We may remember that carbonyls may be protected as sturdy azines and then deprotected by reacting with HOF•CH_3_CN (see ref. [46] and Scheme 18). Dimethylhydrazine, however, will not allow the formation of azine, and its reactions with aldehydes are usually fast and quantitative. When such hydrazones were reacted with less than 3 mole equivalents of HOF•CH_3_CN complex, the appropriate nitrile was obtained, usually with a higher than 90% yield. The reaction is general, and dimethylhydrazones of alkyl-aldehydes (**146**), keto-aldehydes (**147**), or heterocyclic-aldehydes (**148**) produced the corresponding nitriles (**149**–**151**) in a few seconds and with very high yields. It should be emphasized that this transformation did not affect the stereochemistry around the starting aldehyde. Thus, reacting the *N*,*N*-dimethylhydrazone of (1*S*,2*S*,5*S*)-(−)-myrtanal (**152**) and the *N*,*N*-dimethylhydrazone of (R)-(+)-2,2-dimethyl-1,3-dioxolane-4-carboxaldehyde (**153**) with HOF•CH_3_CN for a few seconds resulted in the corresponding nitriles **154** and **155** without any racemization [75] (Scheme 31).

## 4. Attaching the Oxygen Atom of HOF•CH_3_CN to Sulfur and Other Atoms

### 4.1. Reactions with Aliphatic and Aromatic Sulfides and Thiols

**Scheme 32:**
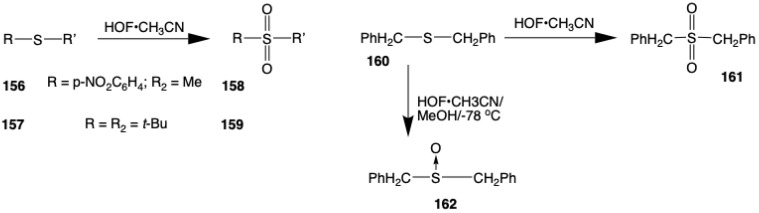
From sulfides to sulfones (and sulfoxide).

As with various π centers discussed above, sulfur has also nucleophilic electrons ready to interact with electrophiles. Indeed, employing the non-polluting HOF•CH_3_CN for oxidizing sulfides directly to sulfones proved to be very efficient. The reaction usually proceeds with quantitative yields at temperatures around 0 °C and in 5–10 min. Examples of such reactions are the aromatic methyl-p-nitrophenylsulfide (**156**) and the aliphatic sterically hindered di-*t*-butyl sulfide (**157**). Both were converted to the respective sulfones **158** and **159.** When only one mole of the HOF•CH_3_CN complex was used with dibenzyl sulfide (**160**), for example, the reaction resulted in a 1:1 mixture of sulfone **161** and starting sulfide. This is further support for the Srnec hypothesis that the oxidizer is a dimer through hydrogen bonding (see ref. [26] and Scheme 27), so it can double-attack the sulfur atom and deposit two oxygen atoms simultaneously. To strengthen this hypothesis, both reagent and sulfide were prediluted with water and methanol to break the hydrogen bond, which keeps the reagent in a form of dimer. The result of this experiment was that three times more sulfoxide **162** was formed than the sulfone **161**. Lowering the reaction temperature to −78 °C produced almost exclusively the sulfoxide **162** [76,77] (Scheme 32).

**Scheme 33:**
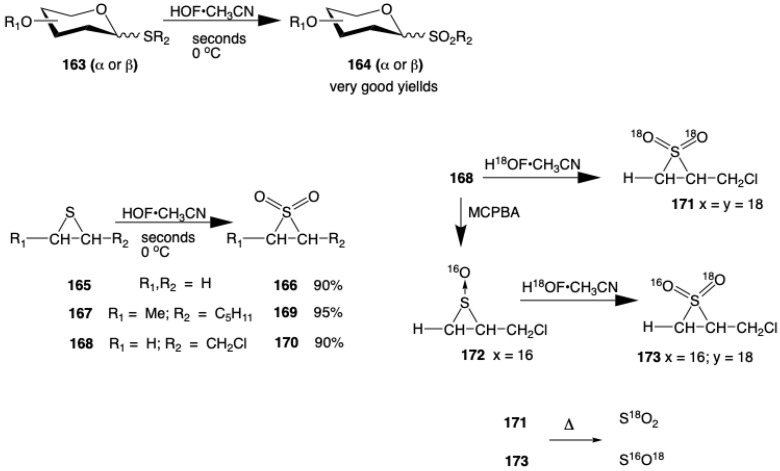
From sulfides to sulfones.

Following the above reactions, Falconer used the oxidizing agent on selected sulfur-containing sugars. The sulfur atoms at the anomeric carbon have been extensively studied either as glycosyl mimics or as synthetic intermediates. The highly oxidized form is of particular interest as a tool for probing biological systems. Many oxidizing agents have been tried, but the authors have not been completely satisfied with any. HOF•CH_3_CN was exactly what they needed, and the reactions of many α- and β-thioglycosides (**163**) turning to the respective **164**s proceeded successfully with high yield and short reaction times [78] (Scheme 33).

Episulfones are a different type of compound, but they have been on the wish list of organic chemists for quite some time. The root of all failures encountered during the attempts at their preparation is the thermal instability of most members of this family. Most oxidizing procedures require some heating, causing a rather easy elimination of SO_2_. Since reactions with HOF•CH_3_CN are fast and do not require any heating, it seemed to be the ideal tool for transferring episulfides to episulfones. Indeed, this was the case. The parent episulfides (thiirane) (**165**) required seconds to produce the thiirane-*S*,*S*-dioxide (**166**), as was also the case for many other episulfides, e.g., 2-methyl-1-pentylthiirane (**167**) or epithiochlorohydrin **168**, which were turned into the respective episulfones **169** and **170**. Since episulfones are quite labile, the reaction offers a unique opportunity to easily and economically synthesize SO_2_ with all possible oxygen isotope variations. Syntheses to this end have been performed in the past for studying many natural and other phenomena associated with SO_2_. Such molecules were obtained by burning sulfur in the environment of expensive ^18^O_2_, a procedure difficult to control with respect to ^16^O_2_ and ^18^O_2_ mixtures. Thus, the epithiochlorohydrin (**168**) was reacted with H^18^OF•CH_3_CN, ending in chloromethylthiirane [all ^18^O]-*S*,*S*-dioxide (**171**). In a parallel reaction, **168** was reacted with MCPBA, resulting in the respective episulfoxides (**172**), followed by a reaction with H^18^OF•CH_3_CN, resulting in [^16^O, ^18^O]-*S*,*S*-dioxide (**173**). Both **171** and **173** could be simply heated to release S^18^O_2_ and S^18^O^16^O [79] (Scheme 33).

**Scheme 34:**
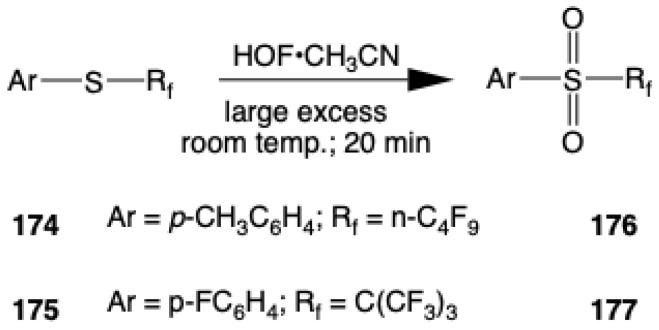
Oxidation of electron-deficient sulfides to sulfones.

Although the oxidation of sulfides with HOF•CH_3_CN is superior in many respects to any other oxidation procedure for this family of compounds, it is still possible to use one of the numerous oxidants developed during the last 150 years to achieve this goal. The situation is different, however, when the sulfur atom in the sulfide is very electron-deficient, as in perfluoroalkylated ones. The oxidation results of such cases are very poor. Only a few methods for converting perfluoroalkyl sulfides to sulfones have been reported, and they typically require reagents like chromic anhydride or highly concentrated H_2_O_2_/trifluoroacetic acid/trifluoroacetic anhydride, and even these potent oxidants are not very efficient. As it turned out, the use of 8 to 10 mole/equivalent excess of HOF•CH_3_CN for 20 min at room temperature proved to be very successful in converting aryl perfluoroalkyl sulfides to sulfones, ArSO2R_f_, some of which are useful second-order, nonlinear optical materials. The following scheme presents a few cases of this transformation, such as perfluoro-*n*-butyl *p*-tolyl sulfide (**174**) and perfluoro-*t*-butyl *p*-fluorophenyl sulfide (**175**) converted to the sulfones **176**, **177** with a higher than 90% yield [80] (Scheme 34).

All the above examples concentrate on the oxidation of various sulfides. HOF•CH_3_CN is also very efficient in oxidizing thiols, either to sulfonic or sulfinic acids, at will. Most aliphatic sulfonic acids are made by the oxidation of thiols, but the aromatic derivatives are usually obtained through sulfonation with sulfuric acid. Still, among the hundreds of aromatic sulfonic acids known in the literature, only a few contain electron-withdrawing groups, and none of those were made by the direct oxidation of thiols or disulfides. Sulfides with either electron-donating or -withdrawing groups on the aromatic ring did not affect the efficiency of the reaction with HOF•CH_3_CN. Thus, for example, pentafluorobenzene sulfonic acid (**178**) and *t*-butylsulfonic acid (**179**) were obtained from the respective thiols **180** and **181** in a few seconds with 90% and 96% yields, respectively. Similar results were obtained when the corresponding disulfides **182** and **183** served as starting materials. The preparation of 5-(trifluoromethyl)-2-pyridinesulfonic acid (**184**) from the corresponding thiol **185** may be of special interest. Even though HOF•CH_3_CN could react with tertiary amines to produce the corresponding *N*-oxides (see Scheme 23), the sulfur atom of the thiol reacted faster than the nitrogen, and **184** was formed in a few seconds with a 91% yield.

**Scheme 35:**
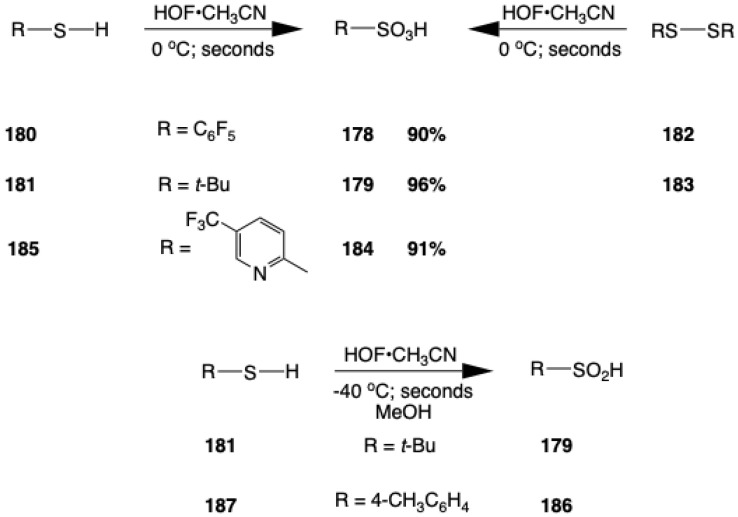
Oxidation of thiols to sulfonic or sulfinic acids.

Several methods are available for the synthesis of sulfinic acids. Mostly, they are indirect and multi-step routes. HOF•CH_3_CN offers a new procedure for one-step preparation by reacting with the same thiols or disulfides but at −40 °C and in the presence of methanol. Thus, the aliphatic *t*-butylsulfinic acid (**179**), as well as the aromatic *p*-toluenesulfinic acid (**186**), were obtained with 75% and 70% yields from the respective thiols **181** and **187**. The low temperature and the methanol, which solvates any excess of the reagent and breaks internal hydrogen bonds, reduce the chance of additional oxidant molecules approaching the sulfur atom, thus preventing further oxidation to sulfonic acids [81] (Scheme 35).

### 4.2. Synthesis of Thiophene-S,S-Dioxides

**Scheme 36:**
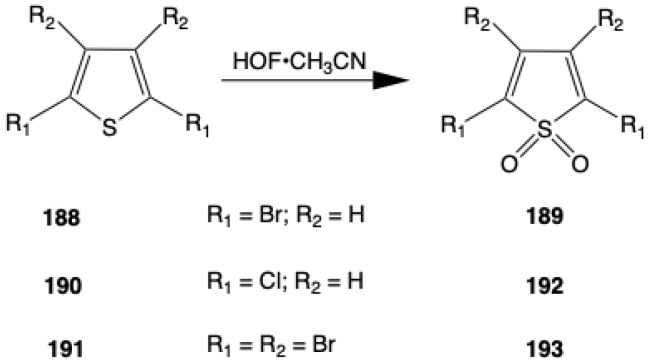
Oxidation of thiophenes to thiophenes-*S*,*S*-dioxides.

Oxidizing the sulfur atom of thiophenes is not an easy task since there are two opposing factors involved. On one hand, the reaction must overcome a substantial aromatic stabilization, which usually requires relatively harsh conditions. On the other hand, these same conditions, high temperatures and prolonged reaction times, are responsible for the resulting nonaromatic diene, (the *S*,*S*-dioxide), which engaged easily in the Diels–Alder and other similar processes. As we have seen in all reactions described above, one of the advantages of HOF•CH_3_CN as a tool for transferring oxygen atoms is its ability to react quickly with any nucleophile under very mild conditions. The oxidation of 2,5-dibromothiophene (**188**) could not be achieved with various peracids, and dimethyldioxirane (DMDO) was only partially successful in forming 2,5-dibromothiophene-*S*,*S*-dioxide (**189**) with a 27% yield after several days of reaction. It took HOF•MeCN only 20 min to form it with a 95% yield. Thiophenes with stronger electron-withdrawing groups are usually beyond the oxidizing power of common oxidizers since under the harsh conditions required, the dioxides undergo various secondary reactions. For example, 2,5-dichlorothiophene (**190**) or tetrabromothiophene (**191**) have never been successfully oxidized with common oxygen transfer agents (including DMDO) to their respective *S*,*S*-dioxides **192** and **193**. By using 4 mole/equivalent (two-fold excess) of HOF•MeCN, **192** and **193** were obtained with a 70% yield [76], [82] (Scheme 36).

**Scheme 37:**
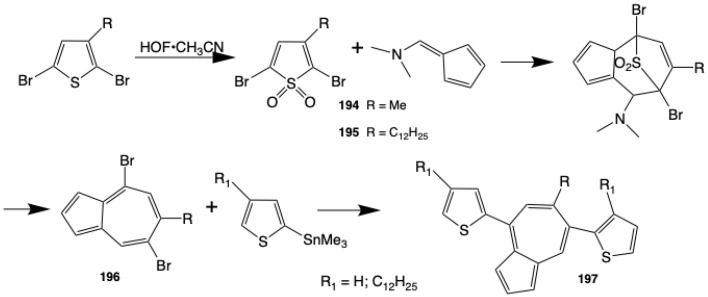
Oxidation of azulenes from thiophenes-*S*,*S*-dioxides.

Hawker used 3-alkyl thiophen *S*,*S*-dioxides (**194**, **195**), obtained by HOF•CH_3_CN, as a source for the 6π + 4π cycloaddition of thiophene-*S*,*S*-dioxides and fulvenes. This reaction offers the synthesis of the azulene nucleus (**196**) in a single step, with the substitution pattern of the seven-membered ring being derived from that of the original thiophene ring. After the crucial oxidative step was completed, several azulene–thiophene oligomers, such as **197**, were prepared, and their physical properties were studied [83] (Scheme 37).

### 4.3. Oligothiophene-S,S-Dioxides

Around the end of the last century, Garnier was one of the first to show that short conjugated thiophene oligomers could serve as efficient active layers in field-effect transistors (FETs) [84]. Since then, the race to develop such organic semiconductors for light-emitting diodes (LEDs) and many other optoelectronic devices has become intense. One of the most important and desired features was reducing the gap between the HOMO-LUMO molecular orbitals. The main synthetic effort for this purpose was centered on the preparation of many derivatives of oligothiophenes (OTs), such as thienylenvinylenes [85], alkyl fluoride oligothiophenes [86], and more. In 1989, a theoretical study suggested that the corresponding oligothiophene-*S*,*S*-dioxides should be the target since their HOMO–LUMO gap might be significantly smaller than the parent OTs themselves [87]. Easier said than done…. Each time such an attempt was undertaken with any orthodox oxidizing agent, only a low yield of partially oxygenated thiophene oligomers was achieved, even after prolonged reaction times [88]. Here is where HOF•CH_3_CN again showed its oxygen-transferring power.

**Scheme 38:**
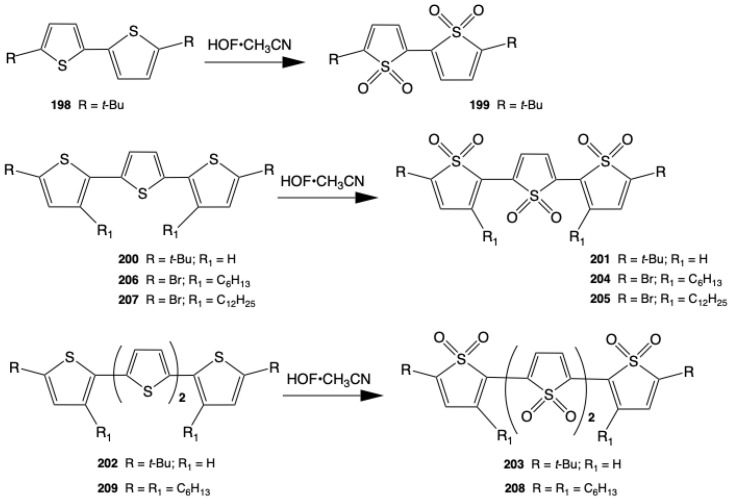
First oligothiophenes [all]-*S*,*S*-dioxides.

5,5′-Dimethyl-[2,2′]bithiophene (**198**) was reacted with this oxidizing reagent at 0 °C for 10 s, resulting in an 85% yield of the bis(*S*,*S*)-dioxide **199**. The methyl groups could be substituted by the bulky tert-butyl ones, providing similar results. Substituting the alkyls with electron-withdrawing bromine atoms did not affect the outcome much. Longer OTs were also treated successfully with the oxidant. It took only one minute to transfer six oxygen atoms to all three sulfur atoms of the 5,5′′-di-tert-butyl-tris-oligothiohene (**200**), affording the tris(*S*,*S*-dioxide) **201** with an 82% yield. This reaction was the first example of a fully oxygenated oligothiophene having more than two thiophene units. Compounds with four thienyl rings, such as 5,5′′′-di-tert-butyl-[2,2′,5′,2′′,5′′,2′′′]-quaterthiophene (**202**), were also successfully oxygenated. In this case, a two-fold excess (16 molar equivalent) of the oxidant and reaction times of up to 40 min at room temperature was required, but eventually, tetrathiophene-[all]*S*,*S*-dioxide **203** was obtained with a 52% yield (Scheme 38). It should be mentioned that the HOMO–LUMO gap of all oxidized *S*,*S*-dioxide was indeed significantly reduced [89]. In addition, all the above *S*,*S*-dioxides were subjected to photophysical and spectroscopic (electronic and Raman) studies guided by density functional theory (DFT). The full oxygenation of the sulfur atoms of oligothiophenes resulted, among other properties, in highlighting the similarity of these compounds to that of oligoenes with a comparable number of π-electrons, although the *S*,*S*-dioxide derivatives were much more thermally stable. Thus, the conclusion was that the oxidation of some or all thiophene rings should be executed to attain materials with lower optical band gaps for solar cell applications [90,91].

The more thiophene-*S*,*S*-dioxide units are in the oligomers, the lower the solubility. Hawker tried to overcome this problem by attaching long aliphatic straight chains (usually hexyls or dodecyl) to the core thiophenes. Specifically, he prepared the dibromo-dihexyl and didodecanyl-terthiophene-[all]-*S*,*S*-dioxides (**204** and **205**) from the non-oxidized oligothiophene **206** and **207**. The bromine atoms made these systems available for subsequent transition metal-catalyzed coupling and could be used as building blocks, leading to more complex conjugated systems. Hawker [92] and Campos [93] prepared even larger soluble oligomers, such as the quarter-thiophene-[all]-*S*,*S*-dioxides, possessing four hexyl groups attached to the end-residing rings (**208**) from the corresponding oxygen-free compound **209.** These works also emphasized the positive effect of lowering the LUMO more than the HOMO level, leading to potentially better electron-accepting properties of the *S*,*S*-dioxide-containing oligomers compared to the aromatic polythiophenes themselves (Scheme 38).

**Scheme 39:**
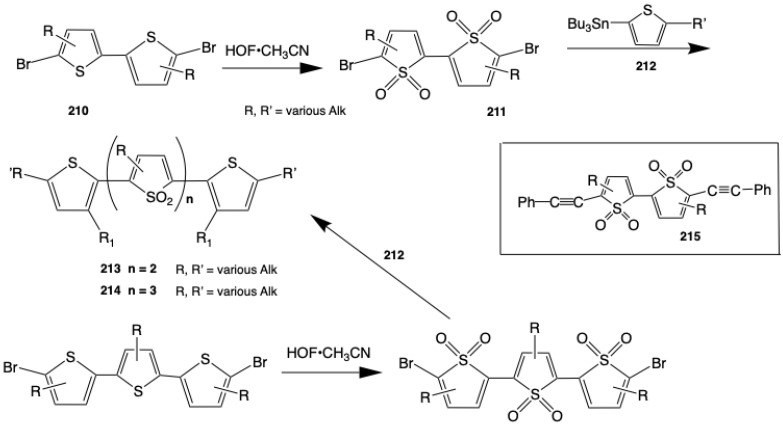
Central *S*,*S*-oxygenated thiophene rings incorporated into oligothiophene chains.

While oligothiophenes [all]-*S*,*S*-dioxides have shown a reduced HOMO–LUMO gap compared to the parent OTs themselves, this is not the only possible configuration. Barbarella suggested that two or three adjacent central *S*,*S*-oxygenated thiophene rings incorporated into oligothiophene chains should have a greater effect on the electron affinity than any other combination of *S*,*S*-oxygenated and non-oxygenated oligothiophenes [94]. A theoretical paper by Casado supported that hypothesis, predicting such polarization [95]. Unfortunately, however, such a theory had never been verified because the relevant materials could not be prepared with the common synthetic tools that organic chemistry offered at the time. An opportunity was presented by HOF•CH_3_CN. Various 5,5′-dibromo-2,2′-bithiophenes (**210**) were oxidized by this reagent in minutes and in quantitative yield to form the corresponding 5,5′-dibromo-2,2′-bithiophene-*S*,*S*,*S*′,*S*′-tetraoxide (**211**). The Stille cross-coupling reaction with 2-(tributylstannyl)thiophene (**212**) afforded the respective quarterthiophene tetraoxide **213** with a 91% yield. A similar procedure afforded quinquethiophene hexoxide (**214**). It should be noted that this procedure also helped create oligomers where instead of terminal thiophene rings, an acetylenic group could be introduced for extending delocalization (**215**). From the optical and electrochemical properties of the compounds described above, it was obvious that the ones with an oxygenated core flanked by non-oxygenated thiophene rings, such as compounds **213** and **214,** display preferable electronic properties, namely considerably reduced band-gaps, higher electron affinities, and higher stabilities towards air, compared with the corresponding non-oxygenated and even [all]-*S*,*S*-oxygenated equivalents. Moreover, these materials exhibit thermal stability, as is evident from their lack of decomposition beneath their melting point temperatures [96] (Scheme 39).

**Scheme 40:**
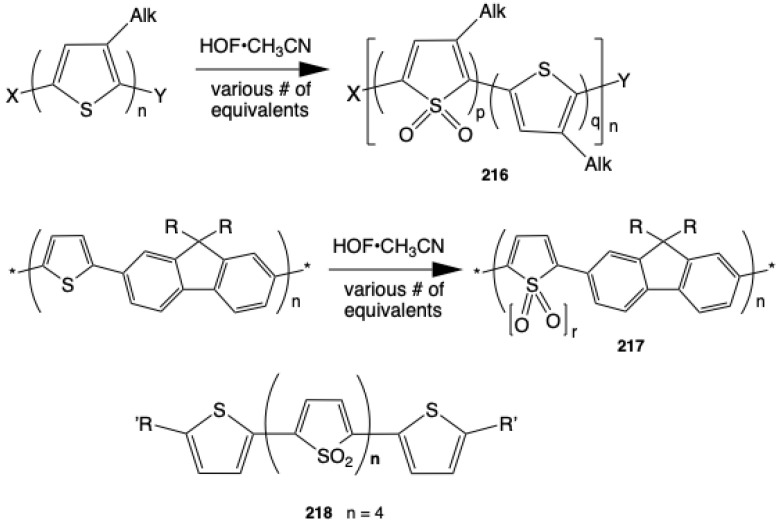
Various co-oligomers of oxidized and non-oxidized thiophenes.

Campos reached similar conclusions from oligothiophene-*S*,*S*-dioxo-containing materials, which he made by treating polymeric chains of polythiophenes with HOF•CH_3_CN, achieving various degrees of oxidation. Thus, he prepared co-oligo-alkyl-thiophene/thiophene-*S*,*S*-dioxide (**216**) and co-poly(9,9-dioctyl-9H-fluorene/thiophene-*S*,*S*-dioxide (**217**) and measured their spectral properties in relation to the number of oxidized units in the polymer, which vary proportionally to the number of the reagent’s equivalents used [97]. In addition, Campos prepared the sexythiophene octaoxide, where the four middle oxidized rings were flanked by two thiophene units (**218**). He used a scanning tunneling microscope (STM)-based break-junction (STM-BJ) technique to provide a means of probing the fundamental electronic properties at the single-molecule level rather than in the bulk material. He also performed thermopower measurements with two electrodes at different temperatures and found that the dominant charge carriers switch from holes to electrons when the number of oxidized monomers in the molecular backbone increases. This underlines the promise of the oxidized oligothiophenes as a powerful electron-deficient motif with a low-lying LUMO to tune the electronic properties in bulk materials [98] (Scheme 40).

**Scheme 41:**
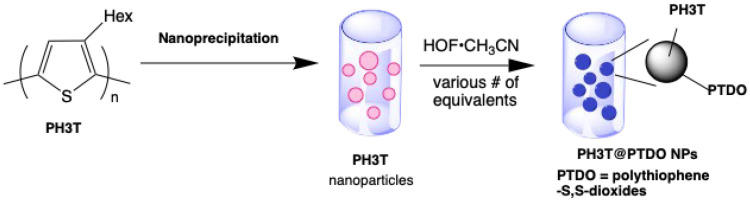
Nanoparticles with positive core and negative shell (n/p nanoparticle).

Di Maria took the above idea of preparing mixed (non-oxygenated/oxygenated) oligomers a step further. She prepared poly(3-hexylthiophene) (P3HT) nanoparticles and exposed them to HOF•CH_3_CN. The result was the formation of core−shell nanoparticles (P3HT@PTDO-NPs), with their structure confirmed by X-ray spectroscopy, indicating the presence of oxygen on the outer shell while the inner shell stays intact. The chemical−physical properties of these nanoparticles were studied. They included absorption and photoluminescence features, electrochemical properties, size, and stability of colloidal solutions, all controlled by the amount of oxygen present. In addition, Kelvin probe measurements show that the coexistence of p- and n-type charge carriers in all types of oxygenated nanoparticles makes them capable of generating and separating charge under illumination. Furthermore, in core−shell nanoparticles, the nano-segregation of the two materials in different regions of the nanoparticles allows for a more efficient charge separation [99] (Scheme 41).

Di Maria also used the same nanoparticles for the efficient photostimulation of retinal neurons thanks to the stronger and longer-lived charge separation in P3HT@PTDO NPs. Initial experiments show it is more effective in the photostimulation of inner retinal neurons than the gold standard P3HT NPs [100].

**Scheme 42:**
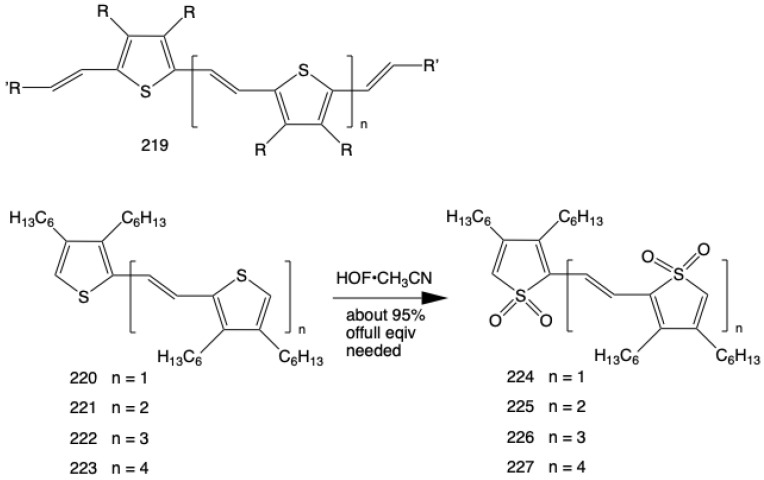
[All]-*S*,*S*-dioxide oligo-thienylenevinylenes.

One of the most important features of oligothiophene-*S*,*S*-dioxides is that the double bonds are in a configuration constituting conjugated dienes easing the move of electrons, which decreases the HOMO-LUMO gap. Naturally, introducing an extra double bond between the thiophene units will yield oligo-thienylenvinylenes with a higher π-electron delocalization. This notion has been examined several times, for example, in the Casado paper with compounds of type **219** [101]. Oxygenated oligo-thienylenevinylene, however, which will further increase the delocalization across the chain, has never been described simply because it could not be made. Using HOF•CH_3_CN created new opportunities. The main potential synthetic obstacle is, of course, the fact that HOF·CH_3_CN is known to epoxidize most double bonds (see Section 2.1 above). The first challenge, therefore, was to transfer oxygen atoms to all sulfur atoms within the chain of the oligo-thiophen vinylenes, leaving the exocyclic double bonds unaffected. It turned out that the sulfur atoms in these oligomers are indeed somewhat more susceptible to attacks by the electrophilic oxygen of the reagent, and if the temperature is kept low and about 95% mole-equivalent of HOF·CH_3_CN is used, the reaction proceeds very satisfactorily. Thus, a series of thiophene-vinylene oligomers (**220**–**223**) were all transformed into their [all] *S*,*S*-dioxides **224**–**227** in short reaction times and with very good yields. Quite a few physical properties were investigated, and it was concluded that these compounds have a chance to be excellent contributors to the organic electronic semiconductor world [102] (Scheme 42).

**Scheme 43:**
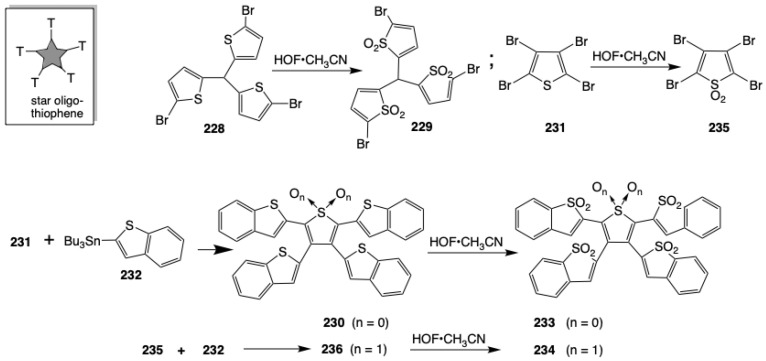
Synthesis of [all]-*S*,*S*-dioxide-“star”-oligothiophenes.

A different configuration of poly-thiophenes consists of star-shaped oligomers (Scheme 43). These compounds have received considerable attention, not only because of their aesthetic appeal. They are promising materials for applications such as active layers in multifunctional organic devices [103], monomers in cross-linked semiconducting polymers [104], and components of conjugated dendrimers [105]. Obviously, it was of interest to find whether these “stars” were, or could be, transformed to their all-*S*,*S*-dioxo derivatives. Both a literature survey and independent efforts to prepare them with peracids or DMDO were completely unsuccessful. The only possible track left was through reactions with the HOF•CH_3_CN complex. Tris(5-bromo-2-thienyl)methane (**228**) [106], with its three thiophene rings attached to a central methyne unit, is a star-shaped oligothiophene. It was reacted at 0 °C, and within 5 min, the novel tris(5-bromo-2-thienyl 1,1-dioxide)methane (**229**) was formed with an 82% yield (Scheme 43). A more complicated star-oligothiophene system is the one with four thiophene rings surrounding a central thiophene unit: 2,3,4,5-tetakis(2-benzothienyl)thiophene (**230**). It was prepared by reacting tetrabromothiophene (**231**) with 2-tributylstannylbenzo[b]thiophene (**232**) under the Stille cross-coupling reaction conditions. Reacting this crowded molecule with HOF•CH_3_CN gave the symmetrical octaoxide (**233**) as a sole product in practically quantitative yield. Any attempt to fully oxidize it to the decaoxide derivative (**234**) by adding an excess of HOF•CH_3_CN was unsuccessful due to the formation of four benzo[all]-*S*,*S*-dioxothiophene groups, where their electron-withdrawing ability reduced the nucleophilic character of the central sulfur atom to the extent of preventing additional electrophilic oxygen attacking it. To solve this problem, an indirect approach had to be adopted. Tetrabromothiophene *S*,*S*-dioxide (**235**) was prepared for the first time by reacting HOF•CH_3_CN with the parent tetrabromothiophene itself (**231**). It was then reacted with 2-tributylstannylbenzo[b]thiophene (**232**) under Stille cross-coupling conditions, forming with a 72% yield a star-thiophene with an already oxidized central ring (**236**). This compound was submitted to oxygenation with HOF•CH_3_CN, eventually forming the desired decaoxide (**234**) with an 88% yield (Scheme 43). It should be mentioned that two prominent physical properties are characteristic of the oxygenated star-thiophenes. The first is their thermal stability, as evident from their melting points, which, in most cases, are above 300 °C, and the second is the very desirable reduction of the HOMO-LUMO energy gap that conspicuously has taken place [107].

**Scheme 44:**
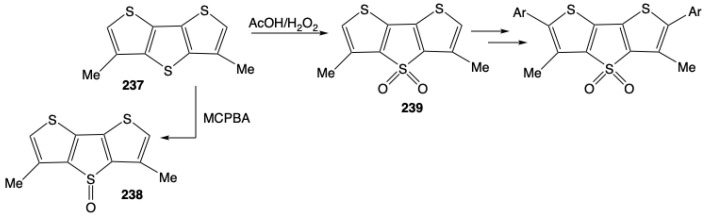
Oxygen transfer to fused thiophenes.

A different type of compound based on the sulfur-containing heterocycles is fused thiophenes. Barbarella thoroughly examined 3,5-dimethyl-2,3′-bis(3-methylthiophene)-dithieno [3,2-*b*;2′,3′-*d*]thiophene (**237**). Treating it with MCPBA resulted in 4-sulfoxide (**238**). Using the more efficient peroxy-acetic acid furnished 4,4-dioxide (**239**) [108] (Scheme 44). It turned out that the last compound, with its “rigid core”, has the highest photoluminescence ever reported for thiophene-based molecules in the solid state. The lumophore is the dithienothiophene-*S*,*S*-dioxide moiety, and the intermolecular interactions (from X-ray diffraction and theoretical calculations) play a major role in determining the solid-state photoluminescence efficiency [109].

However, not a single fused thiophene was made with two of its sulfurs oxidized. How would HOF•CH_3_CN affect these compounds? The 3-bromo-6-nonanylthieno [3,2-*b*]thiophene (**240**) has two thiophene rings, and the reaction resulted in two products, the major being 3-bromo-6-nonanylthieno[3,2-*b*]thiophene 4,4-dioxide (**241**) with an 85% yield, along with a 15% yield of 3-bromo-6-nonanylthieno-[3,2-*b*]thiophene 1,1-dioxide (**242**). Obviously, the regioselectivity of the oxygenation was dictated by electronic factors, and it took place on the more electron-rich sulfur atom. Unlike the case of various oligothiophenes described above, once one sulfur atom was oxygenated, it reduced the nucleophilicity of the neighboring ones, and even HOF•CH_3_CN could not touch it. This was also supported by calculating the Mulliken Charge on the sulfur atoms.

When the fused thiophenes were enlarged as in 2,6-dimethyldithieno[3,2-*b*;2′,3′-*d*]thiophene (**237**) and reacted with HOF•CH_3_CN, the 4,4-dioxide (**239**) was formed with a 90% yield within a few seconds. The latter, however, could not be further oxidized. There were two main reasons for the attack on the central sulfur atom. First, compounds **237** resemble polycyclic aromatic hydrocarbons where the degree of aromaticity is higher on the outer rings, and second, its larger negative charge, as also suggested by DFT calculations, makes it a more potent nucleophile. However, using an excess of HOF•CH_3_CN resulted in equal amounts of **239** and 2,6-dimethyldithieno-[3,2-*b*;2′,3′-*d*]thiophene 1,1,7,7-tetraoxide (**243**) (45% yield each). It was clear that the latter was obtained from the 2,6-dimethyldithieno[3,2-*b*;2′,3′-*d*]thiophene 1,1-dioxide (**244**), which was detected and further oxidized quantitatively to **243**. Such direct oxidation leading to dithienothiophene *S*,*S*,*S*′,*S*′-tetraoxide has never been reported. The reverse selectivity under the excess of the oxidizer was explained by the statistical factors of having two side thiophene units for every central one. Apparently, the electronic influence from the alkyl substitutions of the outer rings has moderated the differences between all the heteroatoms, as also suggested by the DFT calculation.

**Scheme 45:**
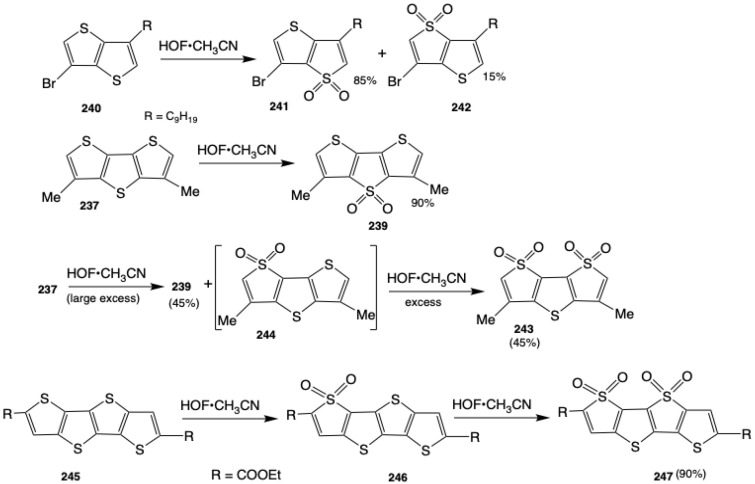
Oxygen transfer to fused thiophenes using HOF•CH_3_CN.

A similar pattern was observed with derivates containing four sequentially fused thiophene rings. When the tetrathienoacence (**245**) was reacted with HOF•CH_3_CN, 2,6-dicarboethoxythieno[3,2-*b*]thieno[2′,3′,4,5]-thieno[2,3-*d*]thiophene 4,4-dioxide (**246**) was formed with a 95% yield. Further oxidation formed the tetraoxide (**247**) with excellent yield [110] (Scheme 45). The UV-vis spectra of most oxidized thieno[2,3-*b*]thiophenes cause a red shift of about 50–72 nm. This indicates a desirable lowering of the HOMO-LUMO energy gap (Scheme 45) [111].

**Scheme 46:**
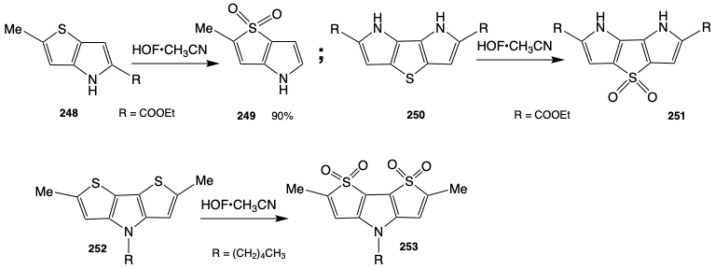
Oxidation of thienopyrroles.

It was of some interest to replace some of the sulfur atoms in the above compounds with nitrogen, as in thienopyrrols, and examine the results of their reaction with HOF•CH_3_CN. Would a differentiation between these two heteroatoms be achieved? What is more, one should investigate the opportunity to have the nitrogen atom attached to alkyl groups to increase solubility, while the sulfur could be transformed into a sulfonyl moiety. This would considerably reduce the HOMO-LUMO band gaps, which is a very desirable feature where various electronic applications are concerned. Results with ethyl 2-methyl-4H-thieno[3,2-*b*]-pyrrole-5-carboxylate (**248**), for example, were encouraging. Reaction with HOF•CH_3_CN for a few seconds at room temperature resulted in the formation of the *S*,*S*-dioxide (**249**) with a 90% yield where none of the double bonds nor the nitrogen atom were affected. With members containing three rings, the results were not that different. Diethyl *4H*,*5H*-thieno[3,2-*b*:4,5-*b*′]dipyrrole-3,6-dicarboxylate (**250**) furnished, after a short treatment with the HOF•CH_3_CN complex, the *S*,*S*-dioxide **251** with high yield. With two thiophene rings engulfing one pyrrole moiety, as in *N*-pentyl 2,6-dimethylditheino[3,2-*b*;2′,3′-*d*]pyrrole (**252**), the reaction with the HOF•CH_3_CN complex formed *N*-pentyl-2,6-dimethyldithieno[3,2-*b*;2′,3′-*d*]pyrrole 1,1,7,7-tetraoxide (**253**) instantaneously. Some of the electronic properties of the products could be deduced from their UV-vis spectra. The oxidation of the sulfur atoms causes up to a 50 nm red shift of their respective maximum absorption (λmax). This indicates that, indeed, a very desirable lowering of the HOMO-LUMO energy gap has taken place (Scheme 46) [112].

### 4.4. Oxidation of Phosphor and Selenium

**Scheme 47:**
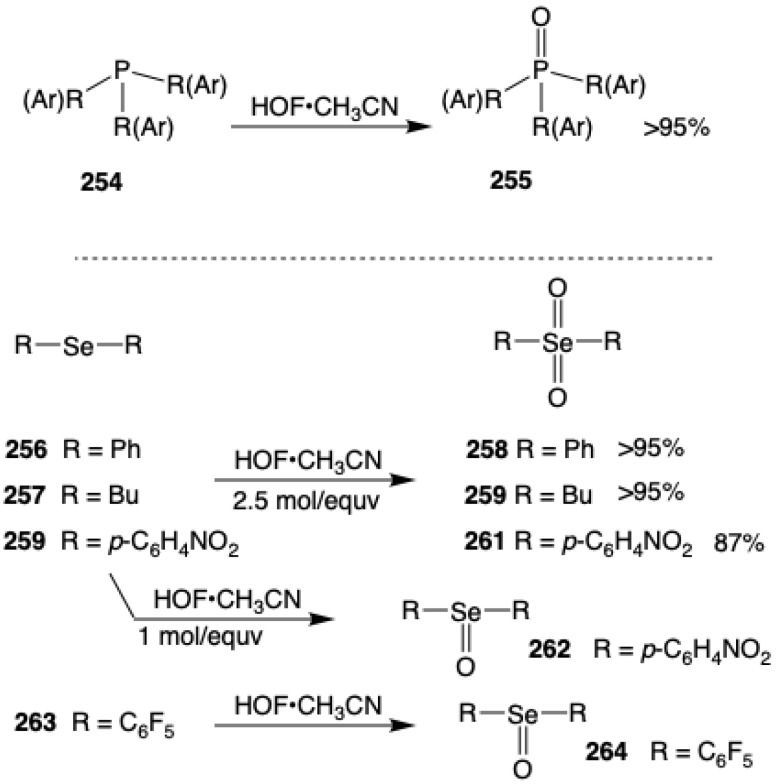
Oxidation of phosphines and selenoides.

The complex of acetonitrile and hypofluorous acid reacts mainly with organic compounds focusing on carbon, nitrogen, and sulfur. Very few works have been devoted to other elements. The two most notable ones are phosphorous and selenium. Shreeve described the known procedure of oxidizing trivalent phosphines to the corresponding phosphine oxides and listed the drawbacks of each method. Reacting various phosphines (**254**) with HOF•CH_3_CN gave phosphine oxides (**255**) (as with other oxidizing agents), but the products were obtained in a very clean form, within 5 min, and with very high yields. Shreeve noted that ^18^O isotope-labeled phosphates, which are very useful molecular probes in biochemistry, could be conveniently obtained by this method if H_2_^18^O instead of the common water was used for the preparation of the H^18^OF•CH_3_CN complex (Scheme 47) [113].

Contrary to the developed selenoxide (R_2_SeO) chemistry, hexavalent selenones (R_2_SeO_2_) have received much less attention, as there is no general method for their preparation [114]. It has been established that the first oxidation of selenide to selenoxide is fast, as opposed to the second sluggish oxidation to selenone, probably because of the dominant preference of selenium for the Se^IV^ oxidation state. For rare cases in which selenones have been available, they were found to be useful intermediates in biologically important processes. The persisting problem, however, was the synthetic barrier that hampered their preparation. HOF•CH_3_CN helped to change this situation. Thus, quite a few diaryl and dialkyl selenides (e.g., Ph_2_Se (**256**) and Bu_2_Se (**257**)) were reacted with a slight excess of the reagent (2.2 mol/equiv) at 0 °C for 3 min, giving the respective selenones **258** and **259** with a higher than 95% yield. Not surprisingly, with strong electron-withdrawing groups such as bis-nitrophenyl selenide **260**, the yield of selenone **261** was somewhat reduced to 87%, and if only 1 mol/equivalent was used, selenoxide **262** was mainly obtained. Selenides with even stronger electron-withdrawing groups, e.g., (C_6_F_5_)_2_Se (**263**), produced only the selenoxide (C_6_F_5_)_2_SeO (**264**), which could not be further oxidized (Scheme 47) [115].

## 5. Conclusions

We hope that the reader and, in fact, the whole chemical community is convinced that HOF•CH_3_CN is a unique tool for transferring oxygen atoms to many molecules. Some of these transformations have already been performed in the past with various orthodox reagents, the difference being speed, temperature, and yield, all in favor of the complex of the hypofluorous acid–acetonitrile. More importantly, however, it can transfer oxygen to many sites that were previously considered impossible to oxidize. Such transformations open many new synthetic possibilities. The fact that at the end of the reaction, the solvent is aqueous acetonitrile, which can be easily recycled, and water-diluted HF—which can be quenched with any base, alumina, and the like—makes it a green reaction. What is still not completely resolved among the chemical community is the mythical fear of F_2_. It is a non-flammable gas, and from a toxicity point of view, it is almost a benign chemical. The American Environmental Group Ltd. found that it is way less toxic than Cl_2_, not to mention Br_2_ [116]. If one uses 10–20% diluted F_2_ in N_2_ (commercial or easy to prepare—see ref. [56]), it cannot cause much harm, even in the case of an accident. We would like to again stress the observation of Appelman [3] and Sharpless [71], who spoke against the “autohypnosis” that started to evolve against fluorine and certain other chemicals, meaning once chemists have accepted some “fact”, most do not bother to confirm it anymore. As a matter of fact, it is easier to work with diluted fluorine compared to many other gases since it does not require special conditions, such as complete dryness, lack of oxygen, and the like. It is also of interest to notice the description of Campos regarding the development of HOF•CH_3_CN: “Rozen aimed to use molecular fluorine in organic synthesis…. This bold decision led to the development of the most powerful oxidizing agent currently known.” [93].

## Data Availability

Not applicable.
